# A Trypanosomatid Iron Transporter that Regulates Mitochondrial Function Is Required for *Leishmania amazonensis* Virulence

**DOI:** 10.1371/journal.ppat.1005340

**Published:** 2016-01-07

**Authors:** Bidyottam Mittra, Maria Fernanda Laranjeira-Silva, Juliana Perrone Bezerra de Menezes, Jennifer Jensen, Vladimir Michailowsky, Norma W. Andrews

**Affiliations:** 1 Department of Cell Biology and Molecular Genetics, University of Maryland, College Park, Maryland, United States of America; 2 Laboratório de Patologia e Biointervenção, CPqGM, FIOCRUZ, Candeal, Salvador, Bahia, Brazil; 3 Faculdade de Medicina, Setor Parasitologia, Universidade Federal do Ceará, Fortaleza, Ceará, Brazil; University of Dundee, UNITED KINGDOM

## Abstract

Iron, an essential co-factor of respiratory chain proteins, is critical for mitochondrial function and maintenance of its redox balance. We previously reported a role for iron uptake in differentiation of *Leishmania amazonensis* into virulent amastigotes, by a mechanism that involves reactive oxygen species (ROS) production and is independent of the classical pH and temperature cues. Iron import into mitochondria was proposed to be essential for this process, but evidence supporting this hypothesis was lacking because the *Leishmania* mitochondrial iron transporter was unknown. Here we describe *MIT1*, a homolog of the mitochondrial iron importer genes *mrs3* (yeast) and *mitoferrin-1* (human) that is highly conserved among trypanosomatids. *MIT1* expression was essential for the survival of *Trypanosoma brucei* procyclic but not bloodstream forms, which lack functional respiratory complexes. *L*. *amazonensis LMIT1* null mutants could not be generated, suggesting that this mitochondrial iron importer is essential for promastigote viability. Promastigotes lacking one *LMIT1* allele (*LMIT1/Δlmit1*) showed growth defects and were more susceptible to ROS toxicity, consistent with the role of iron as the essential co-factor of trypanosomatid mitochondrial superoxide dismutases. *LMIT1/Δlmit1* metacyclic promastigotes were unable to replicate as intracellular amastigotes after infecting macrophages or cause cutaneous lesions in mice. When induced to differentiate axenically into amastigotes, *LMIT1/Δlmit1* showed strong defects in iron content and function of mitochondria, were unable to upregulate the ROS-regulatory enzyme FeSOD, and showed mitochondrial changes suggestive of redox imbalance. Our results demonstrate the importance of mitochondrial iron uptake in trypanosomatid parasites, and highlight the role of LMIT1 in the iron-regulated process that orchestrates differentiation of *L*. *amazonensis* into infective amastigotes.

## Author Summary

Leishmaniasis is a serious parasitic disease that affects 12 million people worldwide, with clinical manifestations ranging from self-healing cutaneous lesions to deadly visceralizing disease. A vaccine is not available, and new and less toxic drugs against this protozoan parasite are urgently needed. Following introduction into vertebrate hosts during a sand fly blood meal, *Leishmania* parasites undergo extensive changes in morphology and metabolism that are critical for adaptation to life inside host macrophages and replication as amastigotes. Earlier studies identified major events that occur during amastigote differentiation, but the signaling mechanism initiating this process remained poorly understood. Previously we demonstrated a novel role for the reactive oxygen species (ROS) H_2_O_2_ in initiating amastigote differentiation, a process proposed to be dependent on iron availability inside the parasite’s mitochondria. In this study we identify LMIT1, a *Leishmania* transmembrane protein that functions as a mitochondrial iron transporter and is conserved in other trypanosomatid protozoan parasites. Reduced LMIT1 expression impairs mitochondrial function in the infective amastigote stage, abolishing parasite virulence. Our findings identify LMIT1 as a promising new drug target, and support the conclusion that iron-dependent ROS signals generated in the mitochondria regulate differentiation of virulent *Leishmania* amastigotes.

## Introduction

*Leishmania spp*., parasitic protozoa from the Trypanosomatidae family, cause a broad spectrum of human diseases collectively referred to as leishmaniasis. An estimated 12 million people worldwide are infected with these parasites, with another 350 million at risk of infection [1]. If not treated, the visceral form of leishmaniasis can cause high mortality. With no efficacious vaccines, the emergence of resistance to existing drugs and the lack of less toxic and affordable treatments, the identification of new targets for therapeutic development is urgently needed.

While alternating between mammalian and sand fly hosts *Leishmania* parasites experience extreme changes in environment [2]. In mammals, *Leishmania* replicate inside acidic parasitophorous vacuoles (PV) of macrophages as oval-shaped amastigotes with a very short flagellum. When ingested by sand flies during a bloodmeal, amastigotes transform into flagellated promastigotes that replicate in the insect’s digestive tract. As they mature, promastigotes cease to replicate, transform into infective metacyclic stages and migrate to the fly proboscis, from where they are reintroduced into a mammalian host during a blood meal. To adapt to these distinct environmental conditions, *Leishmania* undergo extensive morphological changes and metabolic retooling, orchestrated through genome-wide changes in gene expression and post-translational modifications [2, 3]. A shift to pH, temperature, oxygen and nutrient conditions similar to those encountered inside mammalian macrophages has been successfully used to induce promastigote to amastigote differentiation in axenic culture [4, 5]. However, the signaling pathway driving the generation of virulent amastigotes, the most important life cycle form in human infections, is still poorly understood.

Recent developments in redox biology revealed a novel role of reactive oxygen species (ROS), specifically H_2_O_2_, as a signal for differentiation [6]. While the high levels of ROS generated during oxidative stress cause damage to DNA, proteins and lipids, more subtle variations in ROS levels can be involved in signaling pathways that initiate biological processes. Mitochondria-generated ROS is tightly controlled, and its low level modulation has been implicated in regulation of aging, autophagy, immunity and cell fate determination, particularly the transition between cell growth and differentiation [7]. In agreement with these findings, recent work from our laboratory implicated iron-dependent ROS signaling as a trigger for amastigote differentiation in *L*. *amazonensis* [8]. It was suggested that iron deprivation causes “leakage” of electrons from the mitochondrial respiratory chain, generating superoxide radicals that are broken down into H_2_O_2,_ the signaling molecule for differentiation, in a reaction catalyzed by iron dependent superoxide dismutase (FeSOD) [8]. Since all trypanosomatid SODs utilize iron as an essential co-factor, these initial studies could not distinguish between the role of mitochondrial FeSODA [9] and the glycosomal FeSODB (equivalent to cytoplasmic SOD in higher eukaryotes [10]). To test the hypothesis that mitochondria are the major site where iron-dependent H_2_O_2_ responsible for inducing differentiation of infective amastigotes is generated, we investigated the mechanism by which iron enters this organelle in *L*. *amazonensis*.

Regulation of iron levels is critical for maintaining the mitochondrial redox balance. The ability of iron to transition between various oxidation states makes it an ideal redox-active cofactor, which is utilized by virtually all organisms [11]. Iron uptake into mitochondria is essential for the synthesis of two important prosthetic groups, iron-sulfur clusters (ISC) and heme, which are required for the functioning of numerous biochemical processes including the electron transport chain (ETC). However, free ferrous (Fe^++^) iron reacts with oxygen or nitrogen compounds to generate highly toxic reactive radicals via the Fenton reaction. Hence, mitochondrial iron import must be tightly regulated, and coordinated with demand [12]. In this study we identify and functionally characterize the Leishmania Mitochondrial Iron Transporter-1 (LMIT1), a transmembrane protein with mitochondrial localization that has strong similarity with mitoferrin, a demonstrated mitochondrial iron transporter in several organisms. Our results show that LMIT1 is required for normal mitochondrial function, and is a critical determinant of virulence in *L*. *amazonensis*.

## Results

### Identification of *LMIT1* (Leishmania Mitochondrial Iron Transporter 1) as a mitochondrial iron transporter

**Fig 1:**
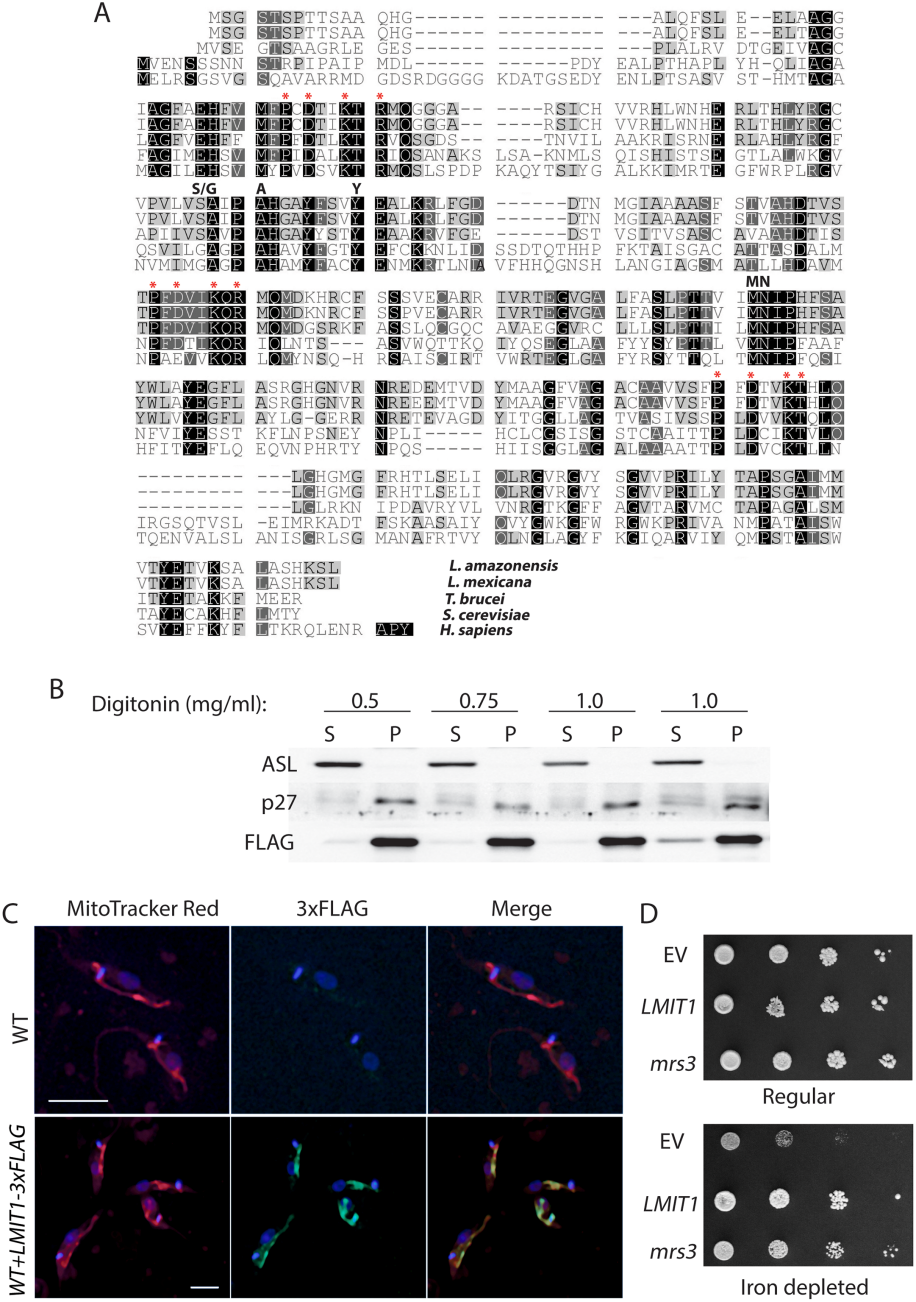
Identification of *LMIT1*, a functional *Leishmania* mitochondrial iron transporter. (A) Multiple sequence alignment showing conservation of functional residues, including the canonical signature sequence motif Px(D/E)xxK/R)x(K/R) (marked with red asterisks), in mitoferrin homologs from *L*. *amazonensis*, *L*. *mexicana* (*LmxM*.*08_29*.*2780*), *T*. *brucei* (*Tb927*.*3*.*2980*) *S*. *cerevisiae* (mrs3) and *H*. *sapiens (*mitoferrin-1) as predicted by ClustalW analysis. Identical and conserved residues highlighted in black, dark and light gray represent 100%, 80% or 60% conservation, respectively. Critical functional residues in substrate contact points are indicated in bold. (B,C) The *Leishmania* LMIT1 protein localizes to mitochondria. (B) Promastigotes expressing LMIT1-3xFLAG were subjected to cellular fractionation following treatment with increasing concentrations of digitonin (as indicated). Antibodies against cytoplasmic adenosuccinate lyase (ASL) and mitochondrial Ldp27 were used to assess separation of cytosolic and mitochondrial proteins in soluble (S) and pellet (P) fractions. The LMIT1-3xFLAG protein was detected using a monoclonal antibody against the FLAG tag. (C) *Leishmania* wild type (WT) and LMIT1-3xFLAG promastigotes were treated with anti-FLAG antibodies followed by a fluorescent secondary antibody (green). Mitochondria were visualized by staining with MitoTracker Red (red), and the localization of LMIT1-3xFLAG tag to mitochondria was assessed by overlaying the images (yellow). Bars = 5 μm. (D) The *Leishmania LMIT1* gene functionally compensates for lack of the mitochondrial iron transporter mrs3 in yeast. *S*. *sacharomyces Δmrs3Δmrs4* was transformed with empty vector pYES2 (EV) or with yeast *mrs3* or *Leishmania LMIT1* constructs. Serial spot growth assays were carried out with transformed yeast cells plated on regular or iron depleted (BPS treated) media in the presence of galactose.

BLAST homology searches of the TriTryp database identified several members of the mitochondrial carrier family as possible homologs of yeast mitochondrial iron transporter protein mrs3 or human mitoferrin-1. The highest identity (E = 3e^-38^) with both human mitoferrin-1 and yeast mrs3 was observed for *LmxM*.*08_29*.*2780* (*L*. *mexicana*), *LinJ*.*29*.*2890* (*L*. *infantum*), *LmJ*.*29*.*2780* (*L*. *major*) and *Tb927*.*3*.*2980* (*T*. *brucei*). Using *Leishmania mexicana* sequence information, the gene fragment *LMIT1*, coding for a 291 amino acid mitoferrin homolog, was amplified from the *Leishmania amazonensis* genome. ClustalW analysis using protein sequences of yeast mrs3, human mitoferrin-1 and their trypanosomatid homologs showed a high degree of conservation (~31% identity and 50% similarity with yeast mrs3; ~33% identity and 48% similarity with human mitoferrin-1) (Fig 1A). Three copies of the Px(D/E)xx(K/)c(K/R) motif and the critical substrate contact point II residues M-N, unique signatures for mitochondrial carrier proteins coordinating iron transport [13], were also conserved in the trypanosomatid proteins. Secondary structure analysis using TMpred (http://www.ch.embnet.org/software/TMPRED_form.html) predicted six transmembrane helices with the signature motifs located at the end of each odd-numbered helix, similar to what was suggested for mrs3 and human mitoferrin [13, 14].

In contrast to mitoferrin homologs from other organisms, all three trypanosomatid MIT1 proteins lacked any identifiable mitochondrial targeting signals. To investigate this issue, promastigotes of *L*. *amazonensis* were transfected with an episomal plasmid encoding LMIT1-3xFLAG, and subjected to sub-cellular fractionation after solubilization with increasing concentrations of digitonin, a method previous validated for generating mitochondrial fractions [15, 16]. Western blot using antibodies against the cytoplasmic enzyme adenylosuccinate lyase [17] and the mitochondrial protein Ldp27 [16] demonstrated that full separation of cytoplasmic and mitochondrial proteins was achieved with 1 mg/ml digitonin (Fig 1B). The LMIT1-3xFLAG protein co-fractionated along with Ldp27 in the mitochondria-enriched fraction. To further confirm the localization of LMIT1-3xFLAG, anti-FLAG antibodies were used to stain the parasites after labeling with the mitochondrial marker MitoTracker Red. As shown in Fig 1C, the tagged version of LMIT1 was targeted to the parasite’s mitochondria.

To investigate the function of *LMIT1*, we performed complementation assays on yeast strains lacking the mitochondrial iron carrier proteins mrs3 and mrs4. This double deletion strain has a severe growth defect under low iron that can be restored by *mrs3* expression [18, 19]. As expected, under limited iron availability (regular growth medium containing the iron chelator BPS) a plasmid driving mrs3 expression rescued the growth of *Δmrs3Δmrs4* yeast more efficiently than the strain transformed with empty pYES2 vector. Importantly, *Δmrs3Δmrs4* yeast cells expressing LMIT1 grew at rates comparable to cells complemented with mrs3 (Fig 1D). Since no differences were observed when all three yeast strains were grown in regular iron-containing medium, this result indicates that the rescue of *Δmrs3Δmrs4* growth in low iron can be attributed to the ability of *L*. *amazonensis* LMIT1 to promote iron import into mitochondria.

### *MIT1* expression is essential for the procyclic, but not the bloodstream form of *T*. *brucei*

Given the high sequence identity between *Leishmania LMIT1 (LmxM*.*08_29*.*2780)* and *T*. *brucei MIT1* (*Tb927*.*3*.*2980*), we performed conditional RNAi-mediated knockdown of the *TbMIT1* gene in *T*. *brucei* to further investigate the importance of this gene in mitochondrial function. Mitochondrial metabolism differs significantly between the two stages of *T*. *brucei* lifecycle. The procyclic form has fully functional mitochondria that house the oxidative phosphorylation machinery involved in ATP generation, primarily through proline catabolism [20]. In contrast, in the bloodstream form ATP generation occurs mainly through the breakdown of glucose within the glycosome, a peroxisome-related organelle [21, 22]. Bloodstream stages lack the respiratory complexes III and IV that are present in procyclics, and although complex I appears to be present, there is no evidence that it contributes to electron transport activity [23]. Thus, the lack of *TbMIT1* expression was predicted to have a stronger impact in procyclics when compared to bloodstream forms.

**Fig 2:**
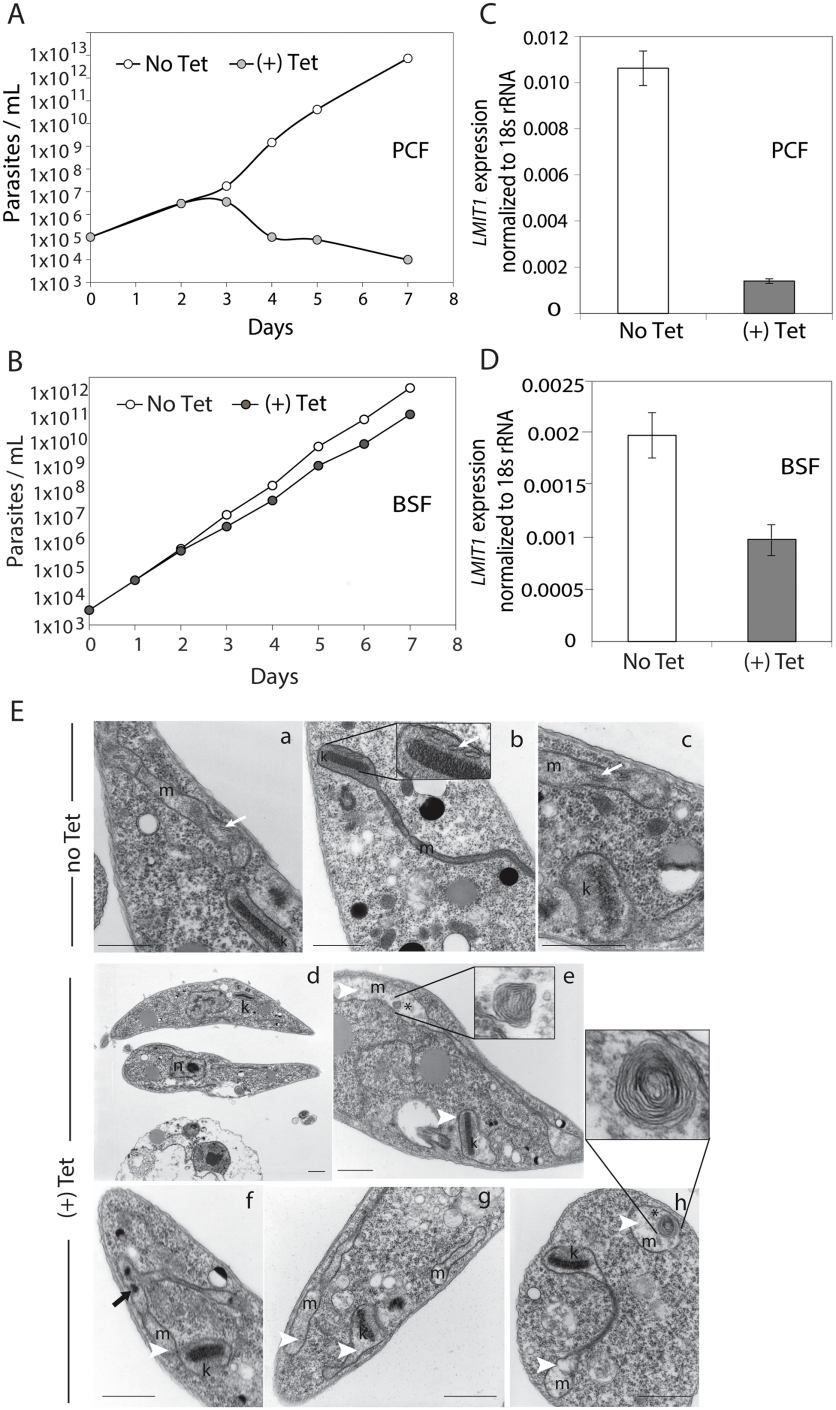
Mitochondria-dependent procyclic forms of *T*. *brucei* require *MIT1* for survival. Procyclic (A) and bloodstream (B) forms of *2T7/MIT1 T*. *brucei* were grown in the absence (No Tet) or presence (+ Tet) of tetracycline (1 μg/ml) to induce dsRNA expression. Cells densities were determined by microscopic counting of samples in duplicate at indicated times. RNA isolated from 1x10^7^ procyclic (C) or blood-stream (D) *2T7/MIT1* cells grown in absence (No Tet) or presence (+Tet) of tetracycline for 48 h were used to quantitate levels of *MIT1* transcript by qPCR. The data show the mean ± SD of the results of three separate experiments. (E) TEM micrographs of *2T7/MIT1* procyclics analyzed on day 3 of the RNAi procedure. Representative micrographs of cells growing in absence (a-c) and presence (d-h) of tetracycline are shown. m, mitochondria; k, kinetoplast; white arrows, normal mitochondrial cristae; black arrows, aggregates inside mitochondria; white arrowheads, enlarged mitochondria; asterisks, membrane whorls inside mitochondria. Bars = 1 μm.

Tetracycline-induced RNAi knockdown of TbMIT1 in procyclics caused an initial decline in parasite growth by day 3, when compared to cultures grown without the drug (Fig 2A). By days 4 and 5 cell numbers had further declined and morphologically abnormal cells with signs of degeneration (such as extensive vacuolation) were increasingly abundant (Fig 2A and 2Ed). Estimation of *TbMIT1* transcript levels by quantitative real time PCR (qPCR) confirmed a reduction of >90% 48 h after tetracycline induction (Fig 2C). By day 3 major changes could also be seen in the ultrastructure of mitochondria, with swelling, lower electron density and loss of cristae (Fig 2Ee–2Eh) evident when compared to mitochondria of uninduced cells (Fig 2Ea–2Ec). The mitochondria of tetracyclin-induced procyclics also frequently contained dark electron dense aggregates and membrane whorls that were previously described as disrupted cristae associated with dysfunctional mitochondria [24]. No obvious abnormalities were noticed in the ultrastructure of other organelles such as the nucleus, endoplasmic reticulum, flagellum, and acidocalcisomes, suggesting that disruption of MIT1 function specifically affected the mitochondria of *T*. *brucei* procyclic forms. In contrast, no significant difference in growth rate was observed between tetracycline-induced and un-induced bloodstream forms subjected to the same RNAi procedure (Fig 2B). Consistent with the limited mitochondrial functionality observed in bloodstream forms, in this life-cycle stage *TbMIT1* transcript levels were significantly lower (>5 fold) than in procyclics (Fig 2C and 2D).

### Deletion of a single *LMIT1* allele impairs growth, alters ROS homeostasis and interferes with the ability of promastigotes to undergo iron-dependent differentiation to amastigotes

Unlike *T*. *brucei*, *Leishmania* has fully functional mitochondria in all life cycle stages [16]. To understand the role of LMIT1, we proceeded to generate *L*. *amazonensis* strains lacking both *LMIT1* alleles through homologous recombination, using knockout constructs carrying drug-resistance gene cassettes flanked by 5’ and 3‘ UTR regions of the *LMIT1* gene (S1A Fig). Replacement of a single *LMIT1* allele was achieved using either the *PHLEO* or *NEO* drug resistance gene cassettes. Allelic integration of the knockout construct into the desired gene locus was confirmed by PCR using specific primers (S1B Fig), and the resulting *LMIT1/Δlmit1* strain showed the expected reduction in *LMIT1* transcripts (S1D Fig). However, repeated attempts to replace the second allele to generate a *LMIT1* null strain were unsuccessful. Even when strains resistant to both Phleomycin and Neomycin were obtained and replacement of the second allele with the targeting drug-resistance marker was confirmed by PCR, amplification of the *LMIT1* gene was still possible with primers specific for its coding region, suggesting the occurrence of gene duplication, as previously reported for other essential *Leishmania* genes [25].

**Fig 3:**
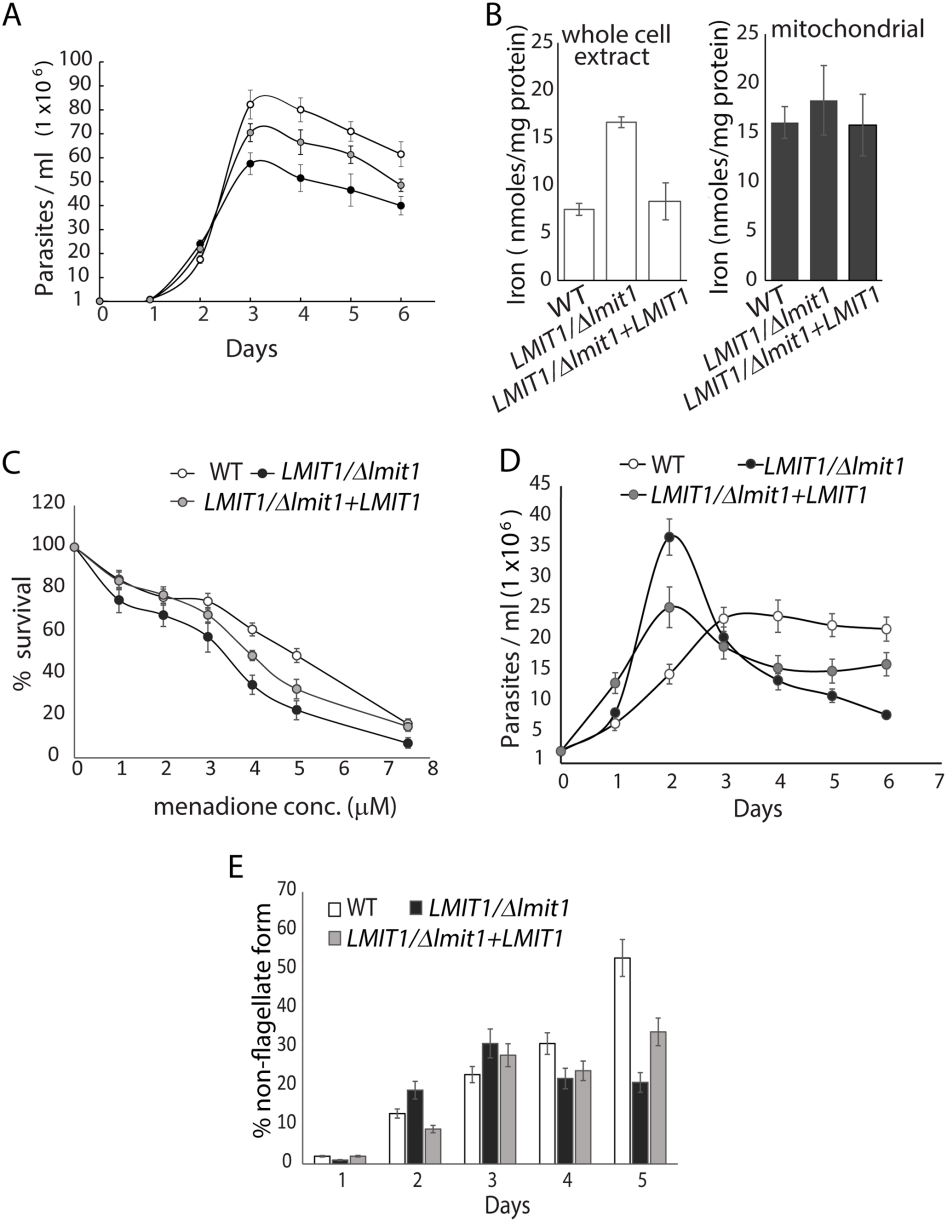
Deletion of one *LMIT1* allele impairs *L*. *amazonensis* promastigote growth, enhances sensitivity to ROS and inhibits amastigote generation triggered by iron deprivation. (A) Growth curves of wild type (WT), single knockout (*LMIT1/Δlmit1*) and complemented single knockout (*LMIT1/Δlmit1+LMIT1)* promastigotes in regular growth medium. (B) The iron content in whole cells and mitochondrial fractions was determined in 4 day-old cultures of wild type (WT), single knockout (*LMIT1/Δlmit1*) and complemented single knockout (*LMIT1/Δlmit1+LMIT1*) promastigotes. The data represent the mean ± SD of three independent experiments. (C) Effect of menadione, an inducer of superoxide generation, on the survival of wild type (WT), single knockout (*LMIT1/Δlmit1*) and complemented single knockout (*LMIT1/Δlmit1+LMIT1*) promastigotes. The parasites were cultured for 48 h in the presence increasing concentrations of mitochondrial superoxide generator menadione and the number of viable cells was determined after staining with FDA. The data expressed as percentage of the number of viable cells in cultures without menadione, represent the mean ± SD of triplicate determinations and are representative of three independent experiments. (D) Growth curves of wild type (WT), single knockout (*LMIT1/Δlmit1*) and complemented single knockout (*LMIT1/Δlmit1+LMIT1)* promastigotes in iron-depleted medium. (E) Fraction of wild type (WT), single knockout (*LMIT1/Δlmit1*) and complemented single knockout (*LMIT1/Δlmit1+LMIT1)* parasites with a rounded morphology and short flagellum after 48 h of culture in iron-depleted medium.

*LMIT1/Δlmit1 L*. *amazonensis* promastigotes showed a normal log phase of growth, but reached stationary phase at a lower cell density (~4–5 x 10^7^/ml) when compared to wild type (~8 x10^7^/ml) (Fig 3A). This defect was partially restored when *LMIT1* was episomally expressed to compensate for the loss of the single *LMIT1* allele (*LMIT1/Δlmit1*+*LMIT1*). This result suggested that LMIT1 plays a role in promastigote survival as they reach the stationary phase of growth, when nutrients (including iron) are depleted from the medium. Ferrozine assays revealed that whole cell lysates of promastigotes that had entered stationary phase (day 4) had a significantly higher iron content when compared to wild type (WT) or complemented *LMIT1/Δlmit1*+*LMIT1* cells, indicating a possible over-accumulation of iron in the cytoplasm due to reduced iron transport into mitochondria. However, at this life-cycle stage (stationary phase promastigotes) no difference was observed in the iron content of mitochondria-enriched fractions from the three strains (Fig 3B).

We previously reported that culture of wild type *L*. *amazonensis* under low iron conditions promotes ROS generation and a reduction in the ability of promastigotes to sustain replication. Under these conditions, the parasites increased their rate of iron uptake, upregulated activity of the ROS detoxification enzyme FeSOD and entered a differentiation pathway that resulted in the generation of amastigote forms. In contrast, parasites lacking the *Leishmania* plasma membrane iron transporter LIT1 failed to upregulate FeSOD activity and accumulated high levels of superoxide, which resulted in massive cell death. These findings led us to suggest that mitochondria is the major site where ROS is generated during cellular stress, and that iron entry into this organelle is essential for activation of the mitochondrial FeSODA and generation of the signaling agonist H_2_O_2_ [8]. To test whether LMIT1 corresponds to the mitochondrial iron transporter involved in this process, we treated promastigotes from wild type, *LMIT1/Δlmit1* and *LMIT1/Δlmit1*+*LMIT1* strains with increasing concentrations of the mitochondrial superoxide generator menadione, and assessed whether loss of one *LMIT1* allele affected the parasite’s ability to survive. *LMIT1/Δlmit1* promastigotes were more sensitive to menadione when compared to wild type, and *LMIT1/Δlmit1*+*LMIT1* showed an intermediate phenotype (Fig 3C).

When grown in iron depleted media, *LMIT1/Δlmit1* promastigotes grew at a faster rate reaching higher concentrations (~3.5 x10^7^/ml) relative to wild type (~1.4 x10^7^/ml) on day 2, followed by a sudden decline in population size after day 3 (Fig 3D). This pattern of initial accelerated growth followed by cell death is similar (but not identical) to what was earlier observed with *L*. *amazonensis* promastigotes lacking the plasma membrane iron transporter LIT1 [8]. Microscopic analysis of viable cells in these iron-depleted cultures revealed that while about 30% (day 4) and 55% (day 5) of the wild type parasites had transformed into amastigote-like rounded forms lacking an evident flagellum, as previously described [8]. In contrast, most cells in *LMIT1/Δlmit1* cultures retained their promastigote-like morphology with long flagella, with only ~20% appearing as round/aflagellate forms on days 4 and 5 (Fig 3E). The complemented strain *LMIT1/Δlmit1+LMIT1* showed an intermediate phenotype in both assays, confirming the ability of LMIT1 to partially rescue the altered responses of *LMIT1/Δlmit1* promastigotes to iron depletion. Collectively, these data suggest that iron import into mitochondria by LMIT1 plays an important role in detoxifying superoxide radicals and in iron/ROS-induced differentiation of promastigotes into infective amastigotes.

### *LMIT1/Δlmit1* promastigotes undergo metacyclogenesis, but fail to replicate intracellularly as amastigotes in macrophages

**Fig 4:**
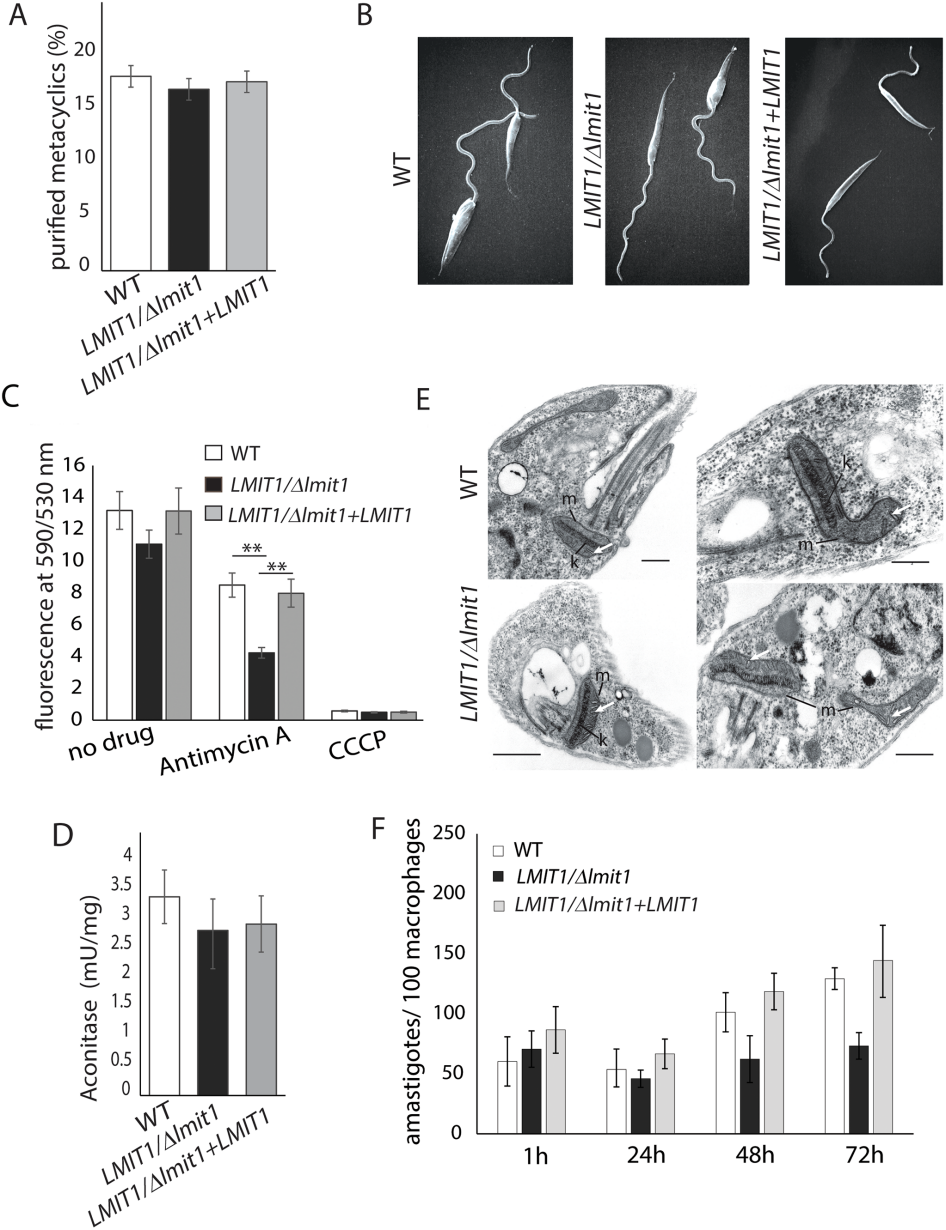
Metacyclogenesis is not affected in *LMIT1* single knockout, but infectivity for macrophages is markedly reduced. (A) A total of 2.5 x 10^8^ wild type (WT), single knockout (*LMIT1/Δlmit1*) or complemented single knockout (*LMIT1/Δlmit1+LMIT1*) parasites from 7-day stationary phase were used to isolate metacyclic forms by selective agglutination of promastigotes with the 3A.1 mAb. The data represents the mean ± SD of the percentage of metacyclic forms recovered in triplicate determinations, and are representative of three independent experiments. (B) SEM images of wild type (WT), single knockout (*LMIT1/Δlmit1*) and complemented single knockout (*LMIT1/Δlmit1+LMIT1*) parasites from 7-day stationary phase cultures. (C) Mitochondrial membrane potential (ΔΨ_m_) was estimated in wild type (WT), single knockout (*LMIT1/Δlmit1*) and complemented single knockout (*LMIT1/Δlmit1+LMIT1*) parasites from stationary phase cultures based on JC-1 uptake, with and without addition of the respiratory chain inhibitor Antimycin A, or the mitochondrial uncoupler,CCCP. The data represents the mean ± SD of three independent experiments (Student’s *t* test **p ≤ 0.01). (D) Aconitase activity was measured in mitochondrial fractions from wild type (WT), single knockout (*LMIT1/Δlmit1*) and complemented single knockout (*LMIT1/Δlmit1+LMIT1*) parasites. (E) TEM of WT (top panels) and *LMIT1/Δlmit1* (lower panels) parasites from 7-day stationary phase cultures. m, mitochondria; k, kinetoplast; white arrows, normal mitochondrial cristae. Bars = 1 μm. (F) BMMs were infected with wild type (WT), single knockout (*LMIT1/Δlmit1*) and complemented single knockout (*LMIT1/Δlmit1+LMIT1*) purified metacyclics and fixed immediately or incubated further for indicated time points, and the number of intracellular parasites was determined microscopically. The data represent the mean ± SD of the results of three independent experiments. The asterisks indicate significant differences in infectivity between WT and *LMIT1/Δlmit1* parasites (Student’s *t* test 48 h, *p* = 0.017; 72 h, *p* = 0.008).

Next, we examined if loss of a single *LMIT1* gene affected differentiation of *L*. *amazonensis* promastigotes into metacyclic forms, the insect stages derived from stationary phase promastigotes that initiate infections in vertebrates. Although *LMIT1/Δlmit1* promastigote cultures reached stationary phase at a lower density (Fig 3A) metacyclogenesis appeared to proceed normally, as indicated by the similar percentage of metacyclic forms isolated from wild type, *LMIT1/Δlmit1* and *LMIT1/Δlmit1+LMIT1* cultures after selective promastigote agglutination with the 3A.1 mAb antibody [26, 27] (Fig 4A). Scanning electron microscopy analysis showed no significant variations in the morphology of metacyclics purified from the three strains (Fig 4B).

To check for possible mitochondrial functional abnormalities in metacyclic promastigotes, stationary phase parasites were treated with JC-1, a dye that exhibits membrane potential-dependent accumulation in mitochondria. A reduction in mitochondrial membrane potential (ΔΨ_m_), a signature of impending mitochondrial failure, is indicated by a decrease in the 530/590 nm fluorescence emission ratio of JC-1. Although a slight reduction in ΔΨ_m_ was observed for *LMIT1/Δlmit1* parasites when compared to the wild type and add-back *LMIT1/Δlmit1+LMIT1* strains, the difference was not statistically significant (Student’s t-test p = 0.47) (Fig 4C). In contrast, a strong ΔΨ_m_ drop was observed for all three strains following treatment with CCCP, a mitochondrial membrane potential uncoupler (Fig 4C). Antimycin A, a complex III respiratory chain inhibitor, caused a partial reduction in ΔΨ_m_ in the wild type and add-back *LMIT1/Δlmit1+LMIT1* strains and a more severe effect in the *LMIT1/Δlmit1*strain (Fig 4C), suggesting that the mitochondrial function of *LMIT1* single knock-out stationary phase promastigotes is more vulnerable to stress. However, the aconitase activity in mitochondria-enriched fractions prepared from wild type, *LMIT1/Δlmit1* and *LMIT1/Δlmit1+LMIT1* stationary phase promastigotes was very similar (Fig 4D), indicating that the functionality of Fe-S cluster proteins remained unaffected at this life-cycle stage. Labeling with the mitochondria specific stains MitoTracker Green (which localizes to mitochondria independently of the membrane potential) and MitoTracker Red CMXRos (that accumulates in mitochondria through a mechanism dependent on membrane potential) showed no distinguishable differences in the shape and volume of mitochondria between stationary phase promastigotes from the three strains (S2 Fig). TEM analysis also showed normal ultrastructure, including the presence of normal cristae, in mitochondria from wild type and *LMIT1/Δlmit1* parasites (Fig 4E). When purified metacyclics were used to infect bone marrow derived mouse macrophages (BMM), *LMIT1/Δlmit1* parasites showed a strong defect in intracellular replication as amastigotes, a defect fully rescued in the *LMIT1/Δlmit1+LMIT1* strain (Fig 4F). This result indicates that high levels of LMIT1 protein are likely to be necessary for the intracellular differentiation of metacyclic promastigotes into amastigotes capable of replicating inside PVs of macrophages.

### *LMIT1/Δlmit1* parasites show defective mitochondrial function when induced to differentiate axenically to infective amastigotes

**Fig 5:**
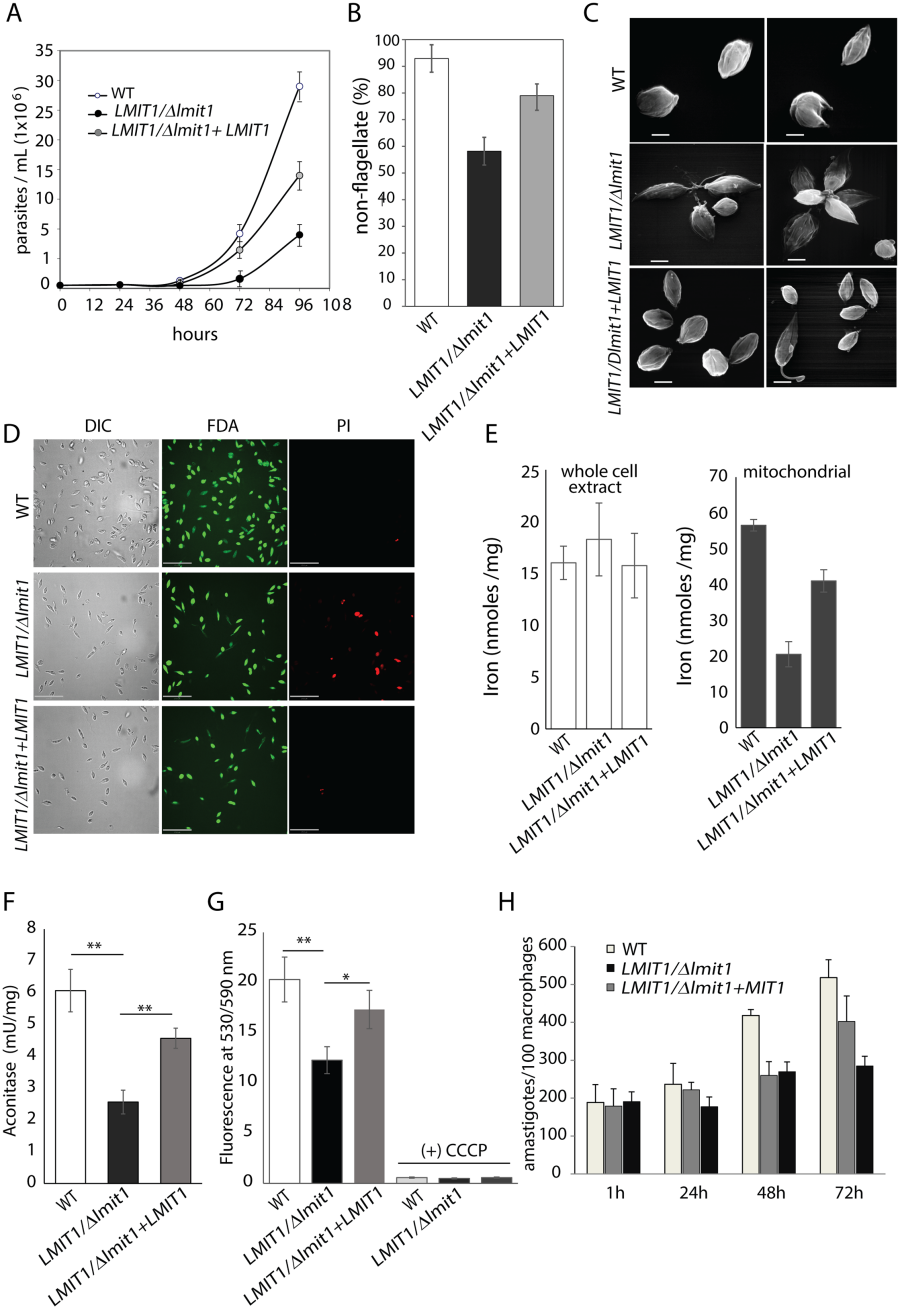
Deletion of one *LMIT1* allele impairs temperature/pH-induced differentiation of promastigotes into axenic amastigotes. Late-log phase promastigotes were washed, resuspended in pH 4.5 at 2x10^5^ parasites/ml, and cultured at 32°C. (A) Numbers of wild type (WT), single knockout (*LMIT1/Δlmit1*) and complemented single knockout (*LMIT1/Δlmit1+LMIT1)* parasites following shift to amastigote medium. The data represent the mean +/- SD of triplicate determinations and are representative of four independent experiments. (B–D) wild type (WT), single knockout (*LMIT1/Δlmit1*) and complemented single knockout (*LMIT1/Δlmit1+LMIT1*) parasites incubated for 48 h in amastigote medium at 32°C were subjected to: (B) Quantification of viable rounded forms with a short flagellum. At least 400 FDA-stained parasites were counted microscopically in each sample. The data represent the mean ± SD of quadruplicate determinations. (C) SEM analysis of the morphology of parasites. Bars = 2 μm. (D) Determination of cell viability and membrane integrity by staining with FDA (green) and PI (red). Bars = 27 μm. (E) The iron content of whole cells and mitochondrial fractions was determined in parasites collected 24 h after induction of axenic differentiation (pH 4.5/ 32°C). Data represents the mean ± SD of three independent experiments (Student’s *t* test *p = 0.037, **p = 0.004). (F) Aconitase activity was determined in mitochondrial fractions from parasites collected 24 h after induction of axenic differentiation (pH 4.5/ 32°C). The data represent the mean ± SD of three independent experiments (**p≤ 0.008). (G) Determination of mitochondrial membrane potential (ΔΨ_m_) with JC-1 with and without prior treatment with the mitochondrial uncoupler CCCP. The data represents the mean ± SD of three independent experiments (Student’s *t* test * *p* = 0.01; **p = 0.002). ((H) Viable amastigotes obtained by temperature/pH- induced axenic differentiation in wild type (WT), single knockout (*LMIT1/Δlmit1*) and complemented single knockout (*LMIT1/Δlmit1+LMIT1*) cultures were tested for their ability to infect BMMs. BMMs were infected and either fixed immediately or after further incubation for 24, 48 or 72 h and the number of intracellular parasites was determined microscopically. The data represent the mean ± SD of triplicate determinations and are representative of more than three independent experiments.

The results discussed above showed that *LMIT1/Δlmit1* promastigotes are defective in iron/ROS-dependent axenic differentiation into amastigotes (Fig 3E), and also cannot replicate as intracellular amastigotes after infecting macrophages (Fig 4F). Next, we examined whether loss of one *LMIT1* allele also affected the axenic differentiation of *LMIT1/Δlmit1* promastigotes into amastigotes using the classical differentiation protocol based on shifting late log phase promastigote cultures (pH 7.4 at 26°C) to conditions mimicking the macrophage intracellular environment (pH 4.5 at 32°C). After an initial lag period of 48 h the wild type parasites replicated steadily in amastigote medium, with a doubling time of ~14 h (Fig 5A). *LMIT1/Δlmit1* cells showed a longer delay (up to 72 h) and very little growth subsequently, reaching cell densities about 6 fold lower than what was observed with the wild type strain at 96 h. At the 48 h time point >90% of the wild type population developed the characteristic amastigote morphology (rounding of the body and shortening of flagella) (Fig 5B). In contrast, only ~55% of viable *LMIT1/Δlmit1* cells underwent a similar morphological change during this period (Fig 5B and 5C). The few *LMIT1/Δlmit1* parasites that succeeded in acquiring the amastigote morphology appeared to be able to replicate axenically, as indicated by the small cell population increase observed by 96 h (Fig 5A). The complemented *LMIT1/Δlmit1+LMIT1* parasites also initiated replication after the first 48 h, and >80% of the parasite population acquired the amastigote morphology (Fig 5A–5C). Staining with reporter dyes indicated that after loss of one *LMIT1* allele *LMIT1/Δlmit1* parasites were still viable by 48 h, based on fluorescein diacetate labeling (Fig 5D). However, by 48 h amastigote membrane integrity started to get compromised, with ~25% of the *LMIT1/Δlmit1* parasites staining positive for propidium iodide (PI), compared to less than 3% in wild type or *LMIT1/Δlmit1+LMIT1* cultures (Fig 5D). To avoid any effects of reduced viability, determinations of iron content and aconitase activity were performed in parasites induced to differentiate for only 24 h, incubation at pH 4.5 and 32°C. Ferrozine assays revealed a significant reduction in the iron content of mitochondrial fractions from *LMIT1/Δlmit1* axenic amastigotes, when compared to wild type and *LMIT1/Δlmit1+LMIT1* parasites (Fig 5E). Consistent with this result, there was also a significant reduction in activity of the Fe-S cluster enzyme aconitase in mitochondrial fractions of *LMIT1/Δlmit1* axenic amastigotes, compared to wild type or the *LMIT1/Δlmit1+LMIT1* complemented strain (Fig 5F). Assessment of mitochondrial membrane potential with the JC-1 dye also revealed a significant drop in ΔΨ_m_ in 48 h *LMIT1/Δlmit1* cultures when compared to wild type, a phenotype that was rescued in the complemented *LMIT1/Δlmit1+LMIT1* strain (Fig 5G). The few *LMIT1/Δlmit1* parasites that appeared able to transform into amastigotes by 96 h were incapable of replicating intracellularly in macrophages when compared to wild type, and this defect was partially reversed in the *LMIT1/Δlmit1+LMIT1* strain (Fig 5H). Taken together, these data indicate that *L*. *amazonensis* amastigotes, either generated intracellularly or axenically, require high levels of LMIT1 expression for maintenance of normal mitochondrial function, replication and survival.

### Inability to upregulate FeSOD activity is associated with mitochondrial damage in amastigotes

**Fig 6:**
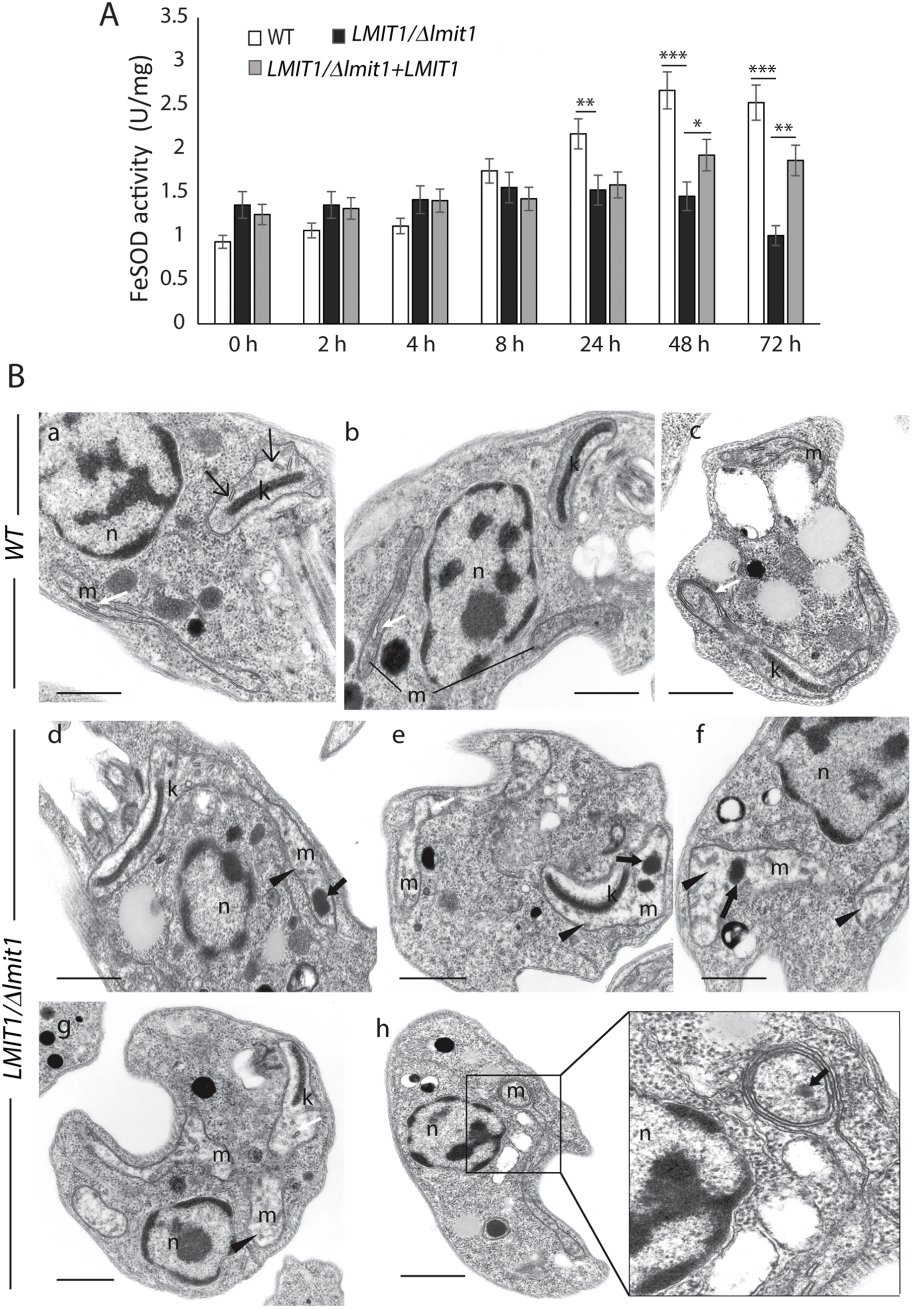
*LMIT1* single knockout parasites induced to differentiate axenically into amastigotes show reduced FeSOD activity and mitochondrial abnormalities. (A) Log phase (~2x10^7^/ml) wild type (WT), single knockout (*LMIT1/Δlmit1*) and complemented single knockout (*LMIT1/Δlmit1+LMIT1*) parasites grown in regular promastigote medium (pH 7.4 / 26°C) were transferred to amastigote media (pH 4.5) and incubated at an elevated temperature (32°C) for 72 h, followed by determination of SOD activity in whole cell extracts. The data represents the mean ± SD of triplicate determinations and are representative of three independent experiments. (Student’s *t* test compared to *WT*: 24 h **p = 0.013, 48 h ***p = 0.0097 and *p = 0.093, 72 h ***p = .0.008 and **p = 0.026). (B) TEM micrographs of wild type (WT) and single knockout (*LMIT1/Δlmit1*) parasites incubated in amastigote media at 32°C for 48 h. (a-c) WT; (d-h) *LMIT1/Δlmit1*. m, mitochondria; k, kinetoplast; white arrows, normal mitochondrial cristae; black arrows, aggregates inside mitochondria; black arrowheads, enlarged mitochondria. Bars = 1 μm.

The higher sensitivity of *LMIT1/Δlmit1* promastigotes to the mitochondrial superoxide generator menadione (Fig 3B) suggested that mitochondrial iron uptake mediated by LMIT1 plays an important role in activation of mitochondrial FeSODA for detoxification of harmful radicals. Given the strong requirement for LMIT1 function in amastigotes, we investigated whether the upregulation of FeSOD activity normally seen during axenic amastigote differentiation [5] was also affected in the *LMIT1/Δlmit1* strain. SOD activity was measured in whole extracts of parasites collected at different time points following the pH and temperature shift. Wild type parasites showed a steady increase in SOD activity up to 48 h, after which the activity remained constant (Fig 6A). On the other hand, FeSOD activity in *LMIT1/Δlmit1* parasites was slightly higher than wild type at the onset of the differentiation process (0 h), but did not increase by 48 h and was lower by 72 h, possibly as a consequence of reduced viability of the cells at that late time point (Fig 5A). The complemented *LMIT1/Δlmit1+LMIT1* strain showed a smaller increase in FeSOD activity when compared to wild type, but reached levels significantly higher than *LMIT1/Δlmit1* parasites by 48–72 h (Fig 6A). The lack of FeSOD upregulation in *LMIT1/Δlmit1* parasites undergoing axenic amastigote differentiation correlated with ultrastructural alterations in mitochondria, which appeared enlarged, with lower electron density and containing dense aggregates, when compared to wild type cells (Fig 6B). Other cellular structures including the nucleus, endoplasmic reticulum, acidocalcisomes, lipid droplets and flagellum appeared normal in *LMIT1/Δlmit1* parasites, providing further evidence that reduction in LMIT1 expression levels has a specific deleterious effect on mitochondria, an effect likely to be related to oxidative damage resulting from reduction in iron availability to activate FeSODA.

### *LMIT1/Δlmit1 L*. *amazonensis* has a strong virulence defect *in vivo*

**Fig 7:**
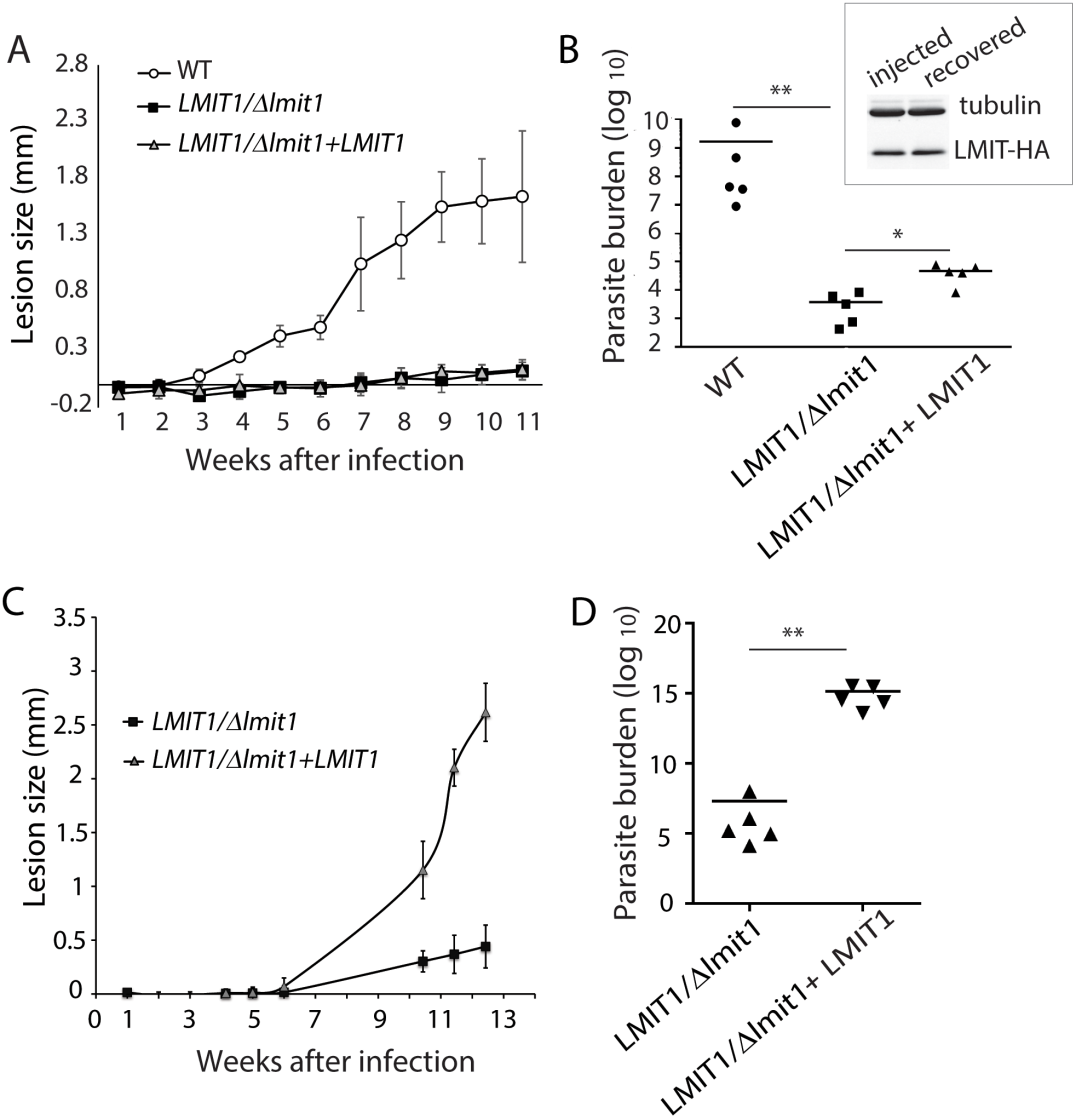
*LMIT1* single knockout metacyclic forms are strongly impaired in virulence for mice. (A) C57BL/6 female mice were inoculated with 1x10^6^ wild type (WT), single knockout (*LMIT1/Δlmit1*) or complemented single knockout (*LMIT1/Δlmit1+LMIT1*) purified metacyclic promastigotes in the left hind footpad and lesion development was measured weekly. The data represent the mean ± SD of 5 mice. (B) The parasite load in footpad tissues was determined 11 weeks after infection (n = 5). **, *p* = 0.009; *, *p* = 0.167 (Student’s t test). Relative levels of episomally expressed LMIT1-HA protein was determined in western blots using 10 μg of whole cell extracts prepared from *LMIT1/Δlmit1+LMIT1* parasites prior to footpad injection (injected) or recovered from lesions post-sacrifice (recovered) and detected with anti-HA antibody. (C) Balb/c female mice were inoculated with single knockout (*LMIT1/Δlmit1*) or complemented single knockout (*LMIT1/Δlmit1+LMIT1*) purified metacyclic promastigotes in the left hind footpad and lesion development was measured weekly. The data represent the mean ± SD of 5 mice. (D) Parasite load in footpad tissues was determined 12 weeks after infection (n = 5). **, *p* = 0.007 (Student’s t test).

To determine how deletion of one *LMIT1* allele affected the ability of *L*. *amazonensis* to infect mice, purified metacyclic promastigotes were injected into footpads of C57BL/6 mice, and cutaneous lesion development was followed for 11 weeks. As expected, the wild type strain induced the formation of progressive lesions during this period. In contrast, no detectable lesions were observed in the mice injected with *LMIT1/Δlmit1* or with *LMIT1/Δlmit1+LMIT1* parasites (Fig 7A). Since lesion size is only an indirect reflection of the actual numbers of parasites in the tissues, after the mice were sacrificed 11 weeks after infection footpads were removed and the parasite load was estimated by the limiting dilution method. Consistent with the marked reduction in lesion development, the parasite burden in animals infected with *LMIT1/Δlmit1* parasites was >10^5^ fold lower than in mice infected with the wild type strain. Moreover, despite the lack of detectable lesions in mice injected with the *LMIT1/Δlmit1+LMIT1* strain, the tissue load data revealed a 10–100 fold, statistically significant increase in parasite numbers for the add-back strain, when compared to *LMIT1/Δlmit1* (Fig 7B). Western blot analysis showed comparable levels of episomally expressed LMIT1-HA protein in *LMIT1/Δlmit1+LMIT1* parasites injected into footpads and subsequently recovered from the tissues, indicating that there was no loss of LMIT1 expression *in vivo* in the complemented strain (Fig 7B, inset). In agreement with this result, when inoculated into the more susceptible Balb/c mouse strain *LMIT1/Δlmit1+LMIT1* metacyclics were more efficient than *LMIT1/Δlmit1* in inducing lesions over a 13 week time period (Fig 7C) and showed a >10^9^ fold higher tissue parasite load (Fig 7D). These results suggest that the LMIT1 protein must be expressed at a minimum threshold level to allow the parasites to overcome the stringent conditions encountered in mouse tissues, and survive and replicate intracellularly as amastigotes. The inability to fully restore virulence through episomal expression of *LMIT1* was not surprising, considering that a lack of robust complementation is a common observation in transgenic *Leishmania* [28, 29].

## Discussion

To our knowledge, this study is the first demonstration that functional mitochondrial iron transporters homologous to mitoferrin are expressed in trypanosomatid parasites. The *Leishmania* LMIT1 protein localizes to the parasite’s mitochondria, rescues the growth defect of a mitoferrin-deficient yeast strain, and its close ortholog *Tb927*.*3*.*2980* in *T*. *brucei* is essential for the survival of procyclic forms but dispensable in bloodstream stages that have rudimentary mitochondria [22]. In agreement with the importance of mitochondrial function in all life cycle stages of *Leishmania* [16, 30], loss of one *LMIT1* allele caused promastigote growth defects, defective amastigote development and severe loss of virulence. Our results are consistent with earlier studies in other systems that identified a direct correlation between rates of mitochondrial iron uptake and increased mitochondrial activity [31–33].

In vertebrates, import of iron into mitochondria occurs via two mitochondrial transporters termed mitoferrin-1 (Mfrn1) and mitoferrin-2 (Mfrn2) [34]. Both carriers are located in the inner mitochondrial membrane. Mfrn1 is expressed at high levels in erythroid cells and is essential for erythropoiesis, while Mfrn2 is ubiquitously expressed and essential for viability in the absence of Mfrn1 [32]. Loss of *Mfrn1* in zebrafish and in mice results in severe reduction of mitochondrial iron in erythroid progenitor cells, and in impaired heme and ISC synthesis [31, 32]. In yeast, the mitoferrin homologs *mrs3p* and *mrs4p* are required for iron import into mitochondria [19], and the poor growth of deletion mutants in low iron can be corrected by expression of vertebrate mitoferrins [18, 31, 35]. *Drosophila* has a single mitoferrin gene that causes partial lethality when deleted and impaired spermatogenesis when expressed at low levels [36]. Our inability to generate viable *L*. *amazonensis LMIT1* null mutants suggests that mitochondrial iron import is also essential in promastigotes of *L*. *amazonensis*.

The reduction in growth rate observed as *Leishmania* promastigotes reach stationary phase may be attributed to increased oxidative stress, resulting from the elevated levels of mitochondrial respiratory chain activity observed at this stage [16]. Thus, an early increase in ROS stress might explain why LMIT1 single knockout promastigotes reach stationary phase prematurely, when compared to wild type. A reduced demand for mitochondrial activity due to cessation of promastigote division may have allowed for sufficient mitochondrial function to support development of metacyclics in *LMIT1* single knockout strains. The subsequent drastic reduction in virulence observed after *LMIT1* single knockout metacyclics were used to infect C57BL/6 mice or bone-marrow macrophages suggests that the mitochondrial iron requirement varies during the *Leishmania* life cycle, being particularly important during differentiation to the amastigote form.

*L*. *amazonensis* promastigotes lacking one *LMIT1* allele were more sensitive to menadione, a drug that causes accumulation of superoxide anion radicals in mitochondria. This phenotype was most likely due to a role of LMT1 in promoting iron entry into mitochondria and activation of FeSODA, the *Leishmania* SOD isoform that catalyzes breakdown of superoxide within mitochondria [9]. Supporting this view, *LMIT1* single knockout strains did not upregulate FeSOD activity when cultivated under axenic conditions that promote promastigote-amastigote differentiation (elevated temperature and acidic pH), a response normally observed in wild type strains [4]. Differentiation into axenic amastigotes was also impaired, and associated with ultrastructural changes in mitochondrial morphology. *LMIT1* single knockout parasites also did not undergo a normal transition from promastigote to amastigote when grown in iron-depleted media, a process that we previously showed to be regulated by iron uptake and FeSOD-dependent H_2_O_2_ generation [8]. Thus, our study strengthens the conclusion that mitochondria represent the major site where superoxide anion is generated and then converted into H_2_O_2_ [6, 7], which can act as a signaling molecule for differentiation of infective amastigote stages [8].

Extensive evidence indicates that access to iron is critical for *Leishmania*, particularly for intracellular amastigotes residing within macrophage PVs. Mutations in Nramp1, a macrophage late endosomal iron transporter, increase host susceptibility to *Leishmania* infections by inhibiting iron removal from PVs [37]. Conversely, *L*. *amazonensis* lacking the plasma membrane ferrous iron transporter LIT1 have a strong defect in intracellular replication and virulence [38]. Infection with *L*. *amazonensis* also inhibits expression of the macrophage iron exporter ferroportin, a process that facilitates amastigote replication by increasing the availability of iron in the cytosol [39]. The present study significantly expands our understanding of *Leishmania* iron acquisition mechanisms, by identifying LMIT1 as a mitoferrin-like transporter that promotes iron entry into the parasite’s mitochondria. This conclusion is strengthened by our results showing that LMIT1 is required for normal functioning of the TCA cycle, the respiratory chain, and for upregulation of FeSOD activity.

The conservation of *MIT1* in several trypanosomatid genomes suggests a common mechanism of mitochondrial iron import despite variations in the pathways by which iron initially enters the parasites. *Leishmania* express the plasma membrane proteins LFR1 (ferric iron reductase—[40]) and LIT1 (ferrous iron transporter—[38]) which promote iron delivery directly into the parasite’s cytosol. In contrast, the bloodstream form of *T*. *brucei* acquires iron by endocytosis of holotransferrin through transferrin receptors (Tfr) in the flagellar pocket. Once internalized, holotransferrin is handled similarly as to what occurs in mammalian cells, with translocation of soluble iron from endosomes to the cytosol through the action of an endosomal ferric reductase and a divalent cation transporter [41]. How *T*. *brucei* procyclics acquire iron is not yet fully understood, since transferrin uptake is absent in these insect forms [42]. The possible existence in *T*. *brucei* procyclics of a *Leishmania*-like machinery for iron uptake has been suggested, based on presence of several putative ZIP domain divalent metal ion transporters, a ferric reductase (LFR1 homolog) and evidence for iron uptake from ferric complexes via a reductive mechanism [43]. Our identification of the highly conserved MIT1 protein suggests that regardless of the mechanism by which iron enters cells, all trypanosomatids import iron from the cytosol into mitochondria utilizing a mitoferrin-like transporter, similarly to what occurs in vertebrates.

*Tb927*.*3*.*2980*, a *T*. *brucei* gene previously identified in a genome-wide mitochondrial carrier inventory and designated as *TbMCP17*, was proposed to correspond to a mitochondrial iron transporter based on its similarity to mitoferrin. However, *TbMCP17* expression and functional information was not available in that initial study [14]. Our inducible RNAi assays now validate the role of *Tb927*.*3*.*2980* (here designated as *TbMIT1*) as the *T*. *brucei* mitoferrin-like mitochondrial iron transporter, by demonstrating that it is essential for viability of the procyclic form. Our assays showed a delay between the loss of *TbMIT1* transcripts (day 2) and appearance of a survival/growth phenotype in *T*. *brucei* procyclics (day 4). This may be attributed to a long half-life of the TbMIT1 protein, or to a gradual accumulation of mitochondrial damage that ultimately led to mortality. This latter scenario is in agreement with the limited role of mitochondria in energy transduction reported for African trypanosomes [44, 45]. *Leishmania*, in contrast, is heavily reliant on mitochondrial activity throughout its life cycle and particularly as amastigotes, which undergo stringent metabolic adjustments to life inside the PV [16, 46]. Intracellular amastigotes are more dependent on the TCA cycle and mitochondrial respiration than on glycolysis for energy production [47]. A functional electron transfer chain was also proposed to be important for maintenance of a neutral intracellular pH (6.5–7.4) in amastigotes replicating in the acidic PV of macrophages (pH 4.5) [3, 48]. Furthermore, the iron-dependent TCA cycle enzyme aconitase is increasingly required to synthesize glutamate, a precursor of the anti-oxidant molecule trypanothione, as transporter-mediated uptake of this amino acid is downregulated in amastigotes [30]. Thus, taken together with the strict iron requirement for activity of FeSODA within mitochondria, these earlier findings are fully consistent with the phenotype we observed in *LMIT1* single knockout mutants, which are partially deficient in that mitochondrial iron transporter.

It will be interesting to determine if the 40% decrease in FeSOD activity that we detected in *LMIT1* single knockout parasites undergoing axenic differentiation can be attributed to changes in mitochondrial FeSODA, without a contribution of the glycosomal FeSODB isoform. In other eukaryotes it is possible to biochemically distinguish between mitochondrial and other cellular SODs, based on the different metals used as cofactors. This distinction is not possible with trypanosomatid enzymes, as both SODs require exclusively iron for activity. However, the mitochondrial alterations we observed in single knockout *LMIT1* parasites incubated at elevated temperature/low pH resemble the previously reported consequences of iron depletion [49] or ROS damage [50], increasing the likelihood that lack of iron to activate mitochondrial FeSODA is a major event underlying the phenotype of *LMIT1* single knockout mutants. Studies in yeast revealed a similar role for mitoferrin in activating mitochondrial FeSOD of bacterial origin [31]. Further supporting this view, superoxide generation generated by mitochondrial hyper-polarization during exposure of *L*. *infantum* to elevated temperature could be countered by overexpression of mitochondrial FeSODA [51]. In this study, by identifying and characterizing LMIT1 as a mitochondrial iron importer, we established a direct connection between iron uptake, mitochondrial redox balance and the development of virulence in *Leishmania*, significantly expanding future options for controlling these serious human infections.

## Materials and Methods

### Ethics statement

All animal work was conducted in accordance with the guidelines provided by National Institutes of Health for housing and care of the laboratory animals and performed under protocol # R-11-73 approved by the University of Maryland College Park Institutional Animal Care and Use Committee on January 14, 2015. The University of Maryland at College Park is an AAALAC-accredited institution.

### *Leishmania* culture

*L*. *amazonensis* (IFLA/BR/67/PH8) was kindly provided by Dr. David Sacks (Laboratory of Parasitic Diseases, NIAID, NIH). Promastigotes were maintained *in vitro* at 26°C in M199 media (pH 7.4) supplemented with 10% heat inactivated FBS, 0.1% hemin (Frontier scientific; 25 mg/ml in 50% triethanolamine), 10 mM adenine (pH 7.5) 5 mM L-glutamine and 5% penicillin-streptamycin [38]. Differentiation of axenic amastigotes was initiated by mixing promastigote cultures (~2-4x10^7^/ml) with equal volumes of acidic amastigote media (M199 conaining 0.25% glucose, 0.5% trypticase and 40 mM sodium succinate pH 4.5) and elevating the temperature to 32°C. Differentiated amastigotes were maintained in amastigote media at 32°C. Parasite viability was ascertained by fluorescent microscopy using fluorescein diacetate (FDA; Sigma-Aldrich) in combination with propidium iodide (PI; Sigma-Aldrich) as described previously [8].

To determine sensitivity to menadione, promastigotes from log-phase cultures (~2x10^7^/ml) were seeded at 4x10^5^/ml with or without increasing concentrations of menadione. Following incubation at 26°C for 48 h, parasites were counted using a hemocytometer.

Iron depleted media was prepared as described earlier [8]. To quantify growth and differentiation in iron-depleted media, mid-log phase *L*. *amazonensis* promastigotes (2x10^7^ / ml) were harvested by centrifugation and resuspended in iron-depleted media at final concentrations of 1x10^6^ or 4x10^6^/ml. Cell growth was measured by microscopic counting of FDA stained cells at different times, as indicated in the experiments. Ability to differentiate was estimated as the percentage of promastigotes with long flagella (undifferentiated) versus rounded forms with short flagella (differentiated) parasites via phase contrast microscopy. At least 200 viable cells per sample were scored.

### *T*. *brucei* culture and RNAi

*Cell culture- T*. *brucei* bloodstream BSF-SM cells and procyclic 29–13 cells [52], which stably express T7 polymerase and Tet repressor, were used for all experiments. Procyclics were cultured in SM9 media containing 15 μg/ml G418 (Gibco) and 50 μg/ml hygromycin (Invitrogen) at 27°C and bloodstream form were maintained at 37°C in HMI-9 medium containing 15 μg/ml G418 as described [53]. Both media were supplemented with 10% tetracycline free FBS (Atlanta Biological).

*Generation of RNAi cell lines-* A 464 bp gene sequence targeting the *T*. *brucei* mitoferrin gene (*Tb927*.*3*.*2980)* for RNAi-mediated knockdown was identified using RNAit software [54] and amplified from *T*. *brucei* genomic DNA using the following oligonucleotides *FD*: *TbMIT1-HindIII* (G*AAAGCTT*AGGAAGTTGCGGGAGATTACA); *RV*: *TbMfn-XbaI* (GA*TCTAGA*ACCTGAAACAAGAACACGGG) (introduced HindIII and XbaI restriction sites are indicated as italicized and underlined nucleotides). The amplified gene fragment was then cloned into p2T7-fla1 [55] by replacing the *fla1* gene using corresponding restriction sites to create the RNAi construct pTbMIT1-KD. The resulting plasmid linearized with NotI enzyme was electroporated into procyclic or bloodstream forms of *T*. *brucei* and stable transfectants (*2T7/MIT1*) were obtained by limiting dilution in 96-well plates with Phleomycin (2.5 μg/ml) for selection as described [53]. Selected clonal lines were then assessed for gene knockdown by qPCR.

*RNAi mediated knockdown-* dsRNA synthesis was induced by the addition of 1 μg/ml tetracycline to cultures of clonal cell lines at 1X10^6^/ml (procyclic) or 1x10^5^/ml (bloodstream) starting concentration. Cells growing in presence or absence of tetracycline were counted daily using a hemocytometer and diluted to the initial starting concentrations. To confirm knockdown of mitoferrin transcripts total RNA isolated from 1x10^7^ cells and qPCR was performed as described [8] using the following primers:

*Tb-Mfn-FD1* (CTCTCTTTGCCCACCACTATTT) and *Tb-Mfn-RV1* (CACCACCCAAGTATGCAAGA) for *TbMIT*; *Tb-18srRNA-FD* (CGGAATGGCACCACAAGAC) and *Tb-18srRNA-RV* (TGGTAAAGTTCCCCGTGTTGA) for *18s rRNA*.

### Generation of *L*. *amazonensis* mitoferrin single knockout cell lines

A 3.3 kbp DNA fragment containing the mitoferrin-like gene (*MIT1*) and its flanking sequences was PCR amplified from *L*. *amazonensis* genomic DNA using primers (*FD Mitoferrin ORF+UTR*- ACAACGCCGTTCGCGACGAT and *RV Mitoferrin ORF+UTR-* ATGCTACGCGGGATTCGCGG) developed based on the *L*. *mexicana* (*LmxM*.*08_29*.*2780*) nucleotide sequence (http://tritrypdb.org) and cloned into pCR2.1-TOPO vector (Invitrogen) to obtain plasmid pLamMIT1, which was then sequenced to obtain the *L*. *amazonensis* specific nucleotide sequence.

To create *LMIT1* deleted mutants, the *L*. *amazonensis LMIT1* open reading frame (ORF) was genetically targeted for removal by homologous recombination using gene deletion constructs containing the Phleomycin resistance gene *ble* (PHLEO) or Neomycin phosphotransferase (NEO). Sequences upstream and downstream of the *MIT1* ORF were cloned using the following primers containing SfiI restriction enzyme sites (underlined) following a previously described method to rapidly generate knock-out constructs [56]: *LMIT1 5’SfiI-A*:*FD*- GAGGCCACCTAGGCCCGGTGCGCCTGTAG and *LMIT1 5’SfiI A*:*RV*- GAGGCCACGCAGGCCGCCCTGCATGCGCG to amplify 5’ sequence; *LMIT1 3’SfiI-A*:*FD–*GAGGCCTCTGTGGCCTCAACGTGAAGCGC and *LMIT1 3’SfiI-A*:*RV–*GAGGCCTGACTGGCCGCAGGCCATCCG for 3’ UTR. Following a four-part ligation using PCR amplified 5’ and 3’ flanking sequences, drug resistance cassettes and the plasmid backbone, positive clones were identified by analyzing SfiI restriction digests of plasmid DNA samples and confirmed by sequencing with specific primers as described [56]. The targeting fragment was liberated by PacI digestion, gel purified and used to transfect *L*. *amazonesis* promastigotes by electroporation as previously described [38]. *LMIT1* single knockout clones were isolated based on the ability of transformants to grow on agar plates containing Phleomycin (50 μg/ml) or neomycin (50 μg/ml) and analyzed by PCR to verify integration of the drug cassette in the desired location.

For generation of a rescue plasmid expressing *LMIT1* with C-terminal hemaglutinin (HA) tag, a two-step PCR amplification strategy was employed. In the first round, a 873 bp fragment of the *MIT1* ORF was amplified from pLamMIT1 with primers (*FD-Mitoferritin HA*: AACCCGGGACAT*ATG*TCTGGCAGCACCTCACC (SmaI site underlined) and *RV-Mitoferritin HA*: AGCGTAGTCTGGGACGTCGTATGGGTAAAGCAAGAGACTCTTGT) that allowed for removal of the endogenous stop codon and introduction of an in-frame HA tag. The PCR product was used as template in a second round amplification using *FD-MIT1* as sense and *RV*:*HA TAG2*: TTGGATCCTTAAGCGTAGTCTGGGACGTCGTATGGTAAGCGTAG (BamHI site underlined) as antisense primers. Similarly, a LMIT1 fusion protein with three FLAG tag copies at the C-terminus was generated by PCR amplifying the *MIT1* gene using *FD-MIT1* as sense and *RV-MIT1-3xFLAG*: TT*GGATCC*CTCACTTGTCATCGTCATCCTTGTAATCCTTGTCATCGTCATCCTTGTAATCCTTGTCATCGTCATCCTTGTAATCCAAGAGACTCTTGT (BamHI site underlined) as antisense primers. The final PCR products were digested with BamHI and SmaI and cloned into pXG-SAT (courtesy of Prof. S. Beverley, Washington University). Transfected *Leishmania* clones were selected in plates containing 50 μg/ml Nourseothricin (Jena Biosciences) and expression of HA or 3xFLAG-tagged mitoferrin was confirmed by immunoblot.

### Complementation of yeast *mrs* mutant strain with *Leishmania* mitoferrin gene

*The Saccharomyces cerevisiae* strain Δ*mrs3*Δ*mrs4-1* (*MAT α his3*Δ*1 leu2*Δ*0 met15*Δ*0 ura3*Δ*0* Δ*mrs3*Δ*mrs4*::*kan MX4)* generated by double deletion of the mitochondrial ion transporter proteins mrs3 and mrs4 in the BY4741 [18] background was used in this assay. Cells were grown in CSM medium that includes yeast nitrogen base, amino acids and glucose. Iron-limited media was prepared by the addition of cell impermeable iron chelator bathophenanthroline disulfonate (BPS) at 50 μM final concentration.

Yeast *MRS3* and codon optimized *L*. *amazonensis LMIT1* gene synthesized for expression in *S*. *cerevisiae* (Integrated DNA Technology) were cloned into pYES2/CT (Invitrogen) with a galactose inducible promoter using BamHI and XhoI sites. Resulting plasmids and the vector alone (as control) were used for transformation of Δ*mrs3*Δ*mrs4-1* using the lithium acetate method [57] and transformants were selected on 2% w/v glucose SC (-Ura) plates. Four to five colonies from each transformation were picked and re-streaked in SC (-Ura) plates containing 2% w/v raffinose as sugar source. Prior to spotting, the cells were cultured in liquid 2% w/v raffinose SC (-Ura) media for 16–18 h for further glucose depletion. Cells were then washed and resuspended into water to a final concentration of 0.2 OD (A_600_). Ten-fold dilutions of each transformant were prepared and 10 μl of each dilution was then spotted on 2% w/v galactose SC (-Ura) plates with or without 50 μM BPS and incubated at 30°C for 4 days prior to imaging.

### Determination of superoxide dismutase activity

Estimation of superoxide activity in whole cell extracts was performed as described earlier [8]. Briefly, 2x10^8^ promastigotes were harvested, washed twice with PBS and resuspended in hypotonic buffer (5 mM Tris–HCl pH 7.8, 0.1 mM EDTA, 5 mM phenylmethylsulfonyl fluoride and 1x complete mini, EDTA-free protease inhibitor cocktail (Roche)) at a final concentration of ~5x10^8^ cells/ml. The parasites were flash-frozen in liquid nitrogen and stored at -80°C freezer until use. To prepare lysates, frozen cells were subjected to three freeze-thaw cycles alternating between liquid nitrogen and a 37°C water bath, lysis was confirmed under the microscope, the lysates were centrifuged at 12,000g for 30 min at 4°C and supernatants carefully collected. Protein contents were determined using a BCA protein assay kit (Thermo Scientific). Superoxide dismutase (SOD) activity in whole cell extracts was measured using the SOD Assay Kit-WST (Dojindo Molecular Technologies, Inc.) according to the manufacturer’s protocol. Standard curves were generated using known concentrations of horseradish superoxide dismutase (Sigma-Aldrich).

### Localization of FLAG-tagged LMIT1 by immunofluorescence microscopy

Promastigotes were incubated with 200 nM MitoTracker Red CMXRos (Invitrogen) for 30 min at 25°C, followed by fixation with 4% paraformaldehyde and attachment to poly L-lysine coated slides (multitest 8-well; MP Biomedicals). After quenching with 50 mM NH_4_Cl for 1 h the cells were permeabilized with PBS 0.1% triton for 15 min, blocked with PBS 5% horse serum and 1% bovine serum albumin (BSA) for 1 h at room temperature (RT) and incubated with mouse anti-FLAG (F1804, Sigma) 1:500 dilution in PBS-1% BSA for 1 h followed by anti-mouse IgG AlexaFluor 488 (InVitrogen) 1:5000 dilution in PBS-1% BSA for 1 h and staining with 2 μg/ml DAPI for 1 h. Slides were mounted with ProLong Gold antifade reagent (Invitrogen), images were acquired through a Deltavision Elite Deconvolution microscope (GE Healthcare) and processed using Volocity Suite (PerkinElmer).

### Assays for mitochondrial activity

Mitochondrial membrane potential (ΔΨ_m_) was measured using the MitoProbe JC-1 assay kit (Invitrogen). Mitochondrial import of the JC-1 lipophilic cationic dye depends on the mitochondrial potential and is independent of the size or shape of the organelle. The green fluorescence (emission 530 nm) of the monomeric dye at low concentration changes to red (emission 590 nm) as it accumulates and forms aggregates. Mitochondrial potential is thus determined by calculating the ratio of 590nm/530nm fluorescence readings, which provides an accurate quantitation of the amount of dye imported into the mitochondria. Promastigote cells (1x10^7^) were incubated with 10 μM JC-1 for 15 min at 27°C, washed and resuspended in PBS. Fluorescence measured at 530 and 590 nm using a SpectraMax M5^e^ microtiter plate reader (Molecular Devices) was used to determine the ΔΨ_m_ (530/590 ratio).

To visualize mitochondria and assess their membrane potential, 1x10^7^ promastigotes were incubated with 0.1 μM MitoTracker Red CMXRos (Invitrogen) and 0.2 μM MitoTracker Green (Invitrogen) for 30 min at 27°C. After washing the cells with PBS, fluorescence for each dye was measured in a fluorimeter per manufacturer’s protocol (MitoTracker Red: excitation at 579 nm and cut-off filter at 599 nm; MitoTracker Green: excitation at 490 nm and cut-off filter at 516 nm). The stained promastigotes were also were placed in glass-bottom dishes (MatTek corporation) for live imaging on a Nikon Eclipse Ti inverted microscope with a 100x NA 1.4 objective (Nikon) equipped with a Hamamatsu C9100-50 camera and mCherry and FITC filters. Acquired images were analyzed with the Volocity Software Suite (PerkinElmer).

### Iron measurements and aconitase activity assay

Mitochondria isolation and fractionation was done as described previously [15, 16] using 5x10^8^ promastigotes from stationary phase culture or parasites undergoing axenic differentiation into amastigotes (pH 4.5 at 32°C temperature). The cells were washed three times with MES (20 mM MOPS pH7.0, 250 mM sucrose and 3mM EDTA) and resuspended in 500 μl of MES supplemented with 1 mg/ml digitonin and protease inhibitor cocktail (Roche). Following 5 min incubation at RT, cell suspensions were centrifuged for 5 min (10,000g at 4°C). The supernatant was collected as the cytoplasmic fraction. The pellet was washed once with MES buffer and used for further analysis as the mitochondrial fraction.

Estimation of intracellular iron content was performed using a colorimetric ferrozine-based assay described previously [8] using 10^8^ parasites. The mitochondrial iron load was determined using mitochondrial-enriched pellets as described earlier. Briefly, whole cells or mitochondrial fractions were lysed with 100 μl of 50 mM NaOH followed by addition of 100 μl 10 mM HCl. 100 μl of iron-releasing solution (prepared by mixing equal volumes of 1.4 M HCl and 4.5% potassium permanganate) was added to the lysates followed by incubation at 60°C for 2 h and addition of 30 μl iron detection reagent containing 6.5 mM ferrozine, 6.5 mM neocuproine, 2.5 M ammonium acetate and 1 M ascorbic acid in water. After 30 min of incubation at RT, 280 μl of each sample was transferred to a 96-well plate, and absorbance at 550-nm wavelength was measured. Iron contents were determined from standard curves generated using ferric chloride solutions of known molarity (0–75 μM).

To measure aconitase activity, the mitochondrial pellet was washed twice with ice-cold 50 mM Tris pH 7.4 buffer containing 0.2 mM sodium citrate, resuspended in 0.2mM sodium citrate and briefly sonicated for 20 s. The suspension was then assayed using the Bioxytech Aconitase-340 assay kit (Precipio Biosciences). Aconitase activity in the mitochondrial lysate converted citrate into isocitrate, which was further converted into α–ketoglutarate by isocitrate dehydrogenase present in the assay mix, with concomitant formation of NADPH from NADP^+^ the rate of which was measured by monitoring the increase in absorbance at 340nm.

### Electron microscopy

For transmission EM (TEM), cells were fixed in 2.5% (v/v) glutaraldehyde in 0.1M sodium cacodylate buffer, pH 7.4 for 60 min and post-fixed with osmium tetroxide in the same buffer for 1 h at RT. Following subsequent standard dehydration steps, the cells were embedded in Spurr’s resin mixture and thin sections were prepared with Reichert Ultracut E. Final images were obtained using Zeiss EM10 CA microscope.

For scanning EM (SEM), parasites fixed in 2.5% (v/v) glutaraldehyde in 0.1M sodium cacodylate buffer, pH 7.4 for 60 min and attached to poly-L-lysine coated coverslips were rinsed briefly with PBS, fixed with 0.1M cacodylate buffer, pH 7.4, treated with osmium tetroxide for 1 h, acetone dehydrated and critical point dried from CO_2_. After sputter coating with Au/Pd, the preparations were imaged in an Amray 1820D scanning electron microscope.

### Quantification of *Leishmania* intracellular growth in macrophages

A total of 1 × 10^5^ BMMs from C57/BL6 mice (Charles River Laboratories) prepared as previously described [58] were plated on glass coverslips in 3 cm dishes 24 h prior to experiments. Infective metacyclic forms were purified from stationary promastigote cultures (7-day old) using the m3Ab monoclonal antibody as described earlier [38]. Attached BMMs were washed with fresh RPMI 1640 and infected with purified metacyclics at 1:5 multiplicity of infection (MOI), or with axenically transformed amastigotes at 1:1 MOI in RPMI supplemented with 10% FBS. After allowing for invasion (1 h for amastigotes and 3h for metacyclics) macrophages were washed three times in PBS and incubated for the indicated times at 34°C. Coverslips were fixed in 4% PFA after 1 (baseline infection) 24, 48, and 72 h of incubation, permeabilized with 0.1% Triton X-100 for 10 min, and stained with 10 μg/ml DAPI for 1 h. The number of intracellular parasites was quantified by scoring the total number of macrophages and the total number of intracellular parasites per microscopic field (100× N.A. 1.3 oil immersion objective, Nikon E200 epifluorescence microscope) and the results were expressed as intracellular parasites per 100 macrophages. At least 300 host cells, in triplicate, were analyzed for each time point. The data were analyzed for statistical significance using an unpaired Student’s t test (*p*< 0.05 was considered significant).

### *In vivo* virulence and parasite load estimation

Six-week-old female C57BL/6 or Balb/c mice (*n* = 5 per group) were inoculated with 1 X10^6^ purified metacyclics [38] from WT, *LMIT1/Δlmit1 and LMIT1/Δlmit1+LMIT1* stationary phase cultures in the left hind footpad in a volume of 50 μl PBS. Lesion progression was monitored weekly by measurements with a caliper (Mitutoyo Corp., Japan), and expressing the data as the difference between the left and right hind footpads. The parasite tissue load was estimated in infected tissue collected from footpads of sacrificed mice11 weeks post infection using a limiting dilution assay [59].

## Supporting Information

S1 Fig Generation of *LMIT1/Δlmit1* mutants. (A) The diagram shows the *LMIT1* gene *(LmxM*.*08_29*.*2780)* flanked by two ORFs of unknown function. Upstream and downstream sequences were targeted for replacing the *LMIT1* alleles with selectable drug markers (DRM) without disruption of upstream and downstream genes. (B) PCR verification of the proper integration of the DRM with primers (red arrows) specific for the drug cassette and genomic DNA downstream of the homologous recombination site. Amplification was performed with forward primer (F) or reverse primer (R) alone or in conjunction (F+R), using genomic DNA isolated from wild type (lanes 1–3) or recombinant colonies (lanes 4–7) Lanes 4 and 5, Neomycin resistant clones B6, C7; lanes 6 and 7, Phleomycin resistant clones B5 and H4). (C) *LMIT1/Δlmit1* promastigotes were transfected with plasmids to drive *LMIT1* episomal expression, and various clones were analysed by Western blot to detect HA-tagged LMIT1 expression in complemented *LMIT1/Δlmit1+LMIT1* lines. (D) qPCR showing reduced *LMIT1* transcript levels in Phleomycin-resistant *LMIT1/Δlmit1* when compared to wild type (*WT*) and complemented *LMIT1/Δlmit1+LMIT1 L*. *amazonensis*.
(TIF)

S2 Fig Mitochondrial function in *LMIT1* single knockout stationary phase promastigotes. The mitochondria of wild type (WT), single knockout (*LMIT1/Δlmit1*) and complemented single knockout (*LMIT1/Δlmit1+LMIT1*) stationary phase promastigotes was stained with MitoTracker Green and MitoTracker Red CMXRos. Passive mitochondrial uptake of MitoTracker Green defines the mitochondrial volume, while uptake of Mitotracker Red CMXRos is ΔΨ_m_-dependent. Merging of the two images indicates active mitochondrial regions in yellow.
(TIF)
