# Supplementary figures and images for: A Trypanosomatid Iron Transporter that Regulates Mitochondrial Function Is Required for Leishmania amazonensis Virulence
Source: PLoS Pathog. 2016 Jan 7;12(1):e1005340. doi: 10.1371/journal.ppat.1005340 (PMC4704735; doi:10.1371/journal.ppat.1005340)

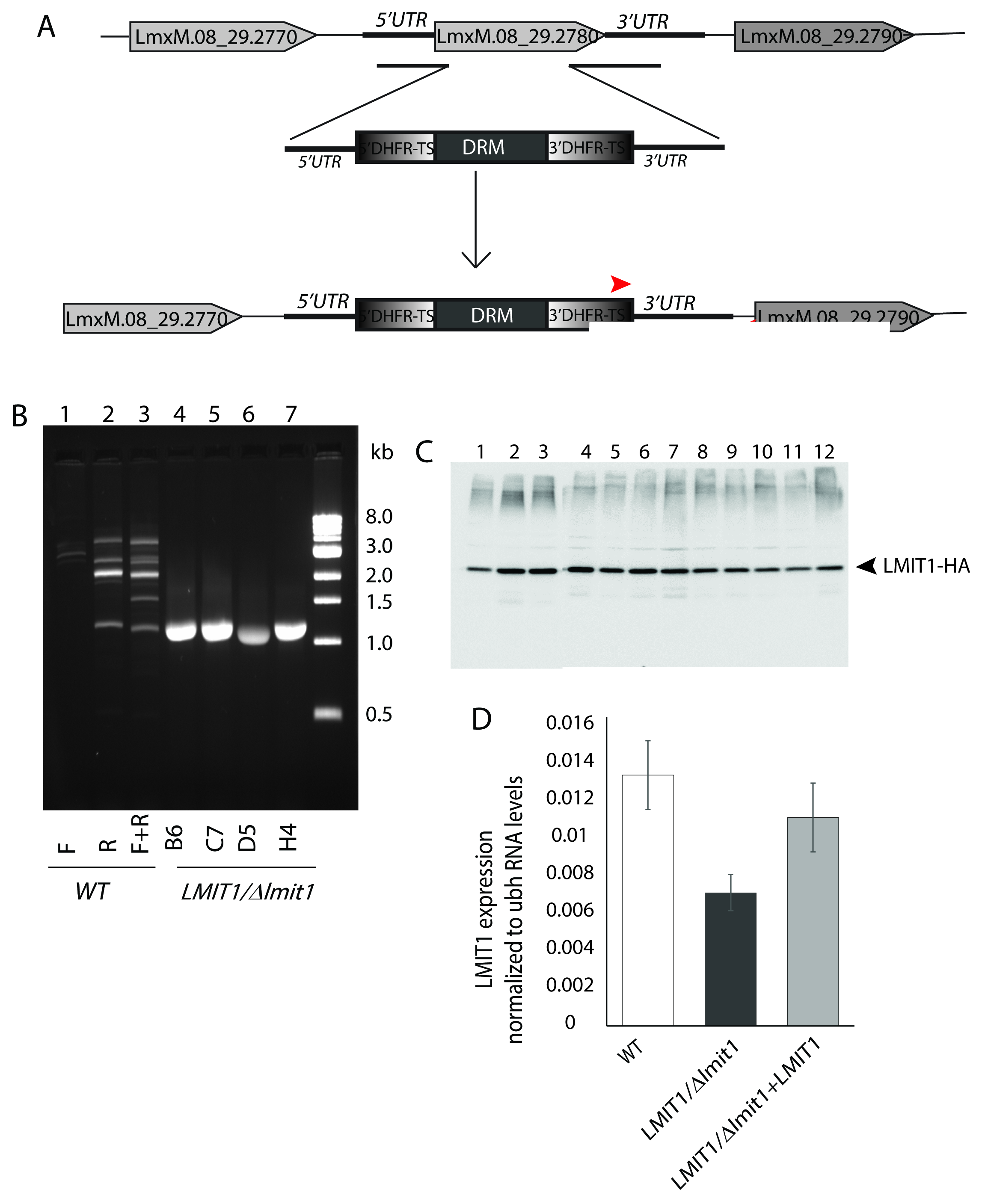

Supplement: S1 Fig — (A) The diagram shows the LMIT1 gene (LmxM.08_29.2780) flanked by two ORFs of unknown function. Upstream and downstream sequences were targeted for replacing the LMIT1 alleles with selectable drug markers (DRM) without disruption of upstream and downstream genes. (B) PCR verification of the proper integration of the DRM with primers (red arrows) specific for the drug cassette and genomic DNA downstream of the homologous recombination site. Amplification was performed with forward primer (F) or reverse primer (R) alone or in conjunction (F+R), using genomic DNA isolated from wild type (lanes 1–3) or recombinant colonies (lanes 4–7) Lanes 4 and 5, Neomycin resistant clones B6, C7; lanes 6 and 7, Phleomycin resistant clones B5 and H4). (C) LMIT1/Δlmit1 promastigotes were transfected with plasmids to drive LMIT1 episomal expression, and various clones were analysed by Western blot to detect HA-tagged LMIT1 expression in complemented LMIT1/Δlmit1+LMIT1 lines. (D) qPCR showing reduced LMIT1 transcript levels in Phleomycin-resistant LMIT1/Δlmit1 when compared to wild type (WT) and complemented LMIT1/Δlmit1+LMIT1 L. amazonensis. (TIF) [file ppat.1005340.s001.tif]

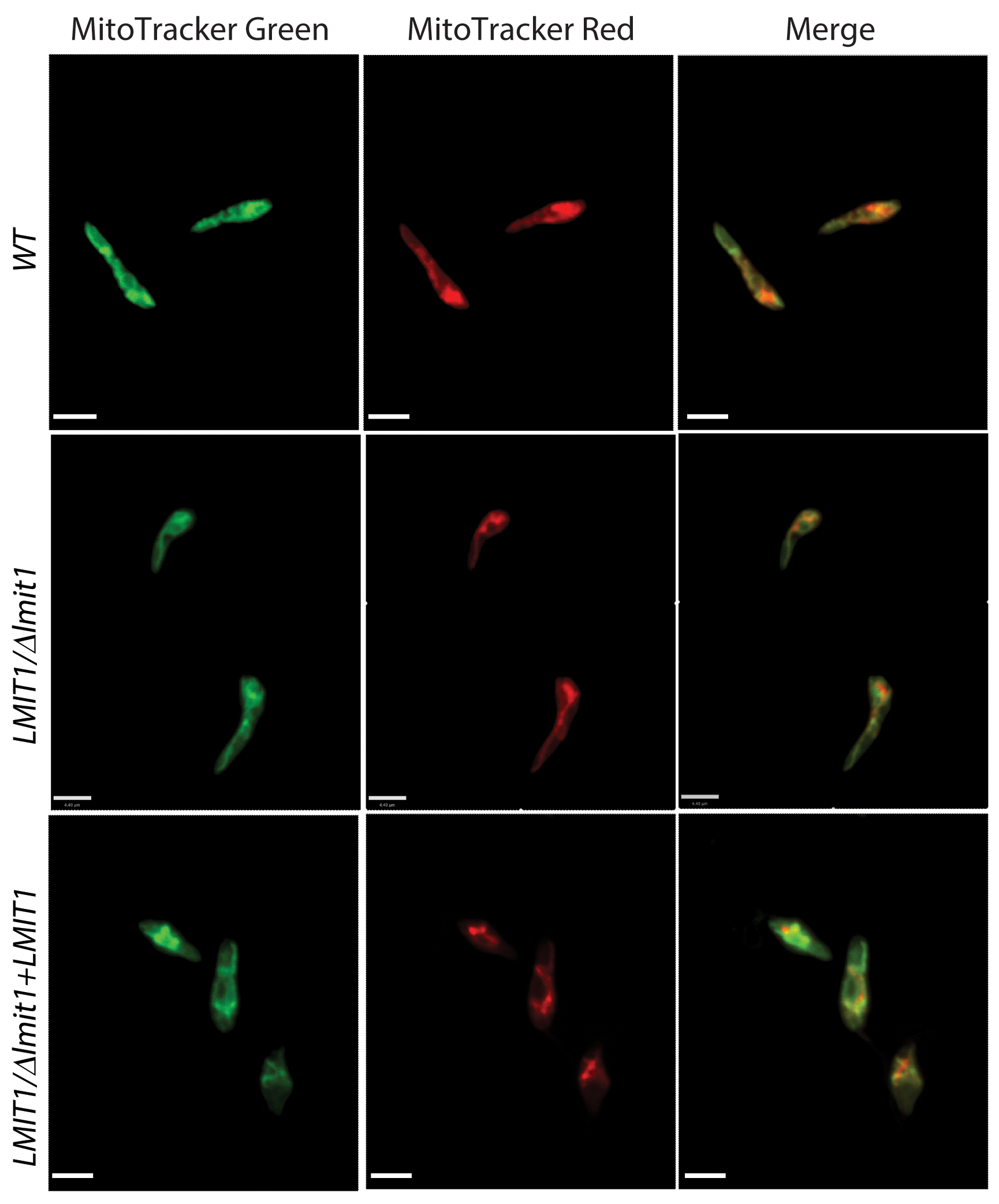

Supplement: S2 Fig — The mitochondria of wild type (WT), single knockout (LMIT1/Δlmit1) and complemented single knockout (LMIT1/Δlmit1+LMIT1) stationary phase promastigotes was stained with MitoTracker Green and MitoTracker Red CMXRos. Passive mitochondrial uptake of MitoTracker Green defines the mitochondrial volume, while uptake of Mitotracker Red CMXRos is ΔΨm-dependent. Merging of the two images indicates active mitochondrial regions in yellow. (TIF) [file ppat.1005340.s002.tif]
